# DNA hydroxymethylation age of human blood determined by capillary hydrophilic-interaction liquid chromatography/mass spectrometry

**DOI:** 10.1186/s13148-015-0109-x

**Published:** 2015-07-23

**Authors:** Jun Xiong, Han-Peng Jiang, Chun-Yan Peng, Qian-Yun Deng, Meng-Dan Lan, Huan Zeng, Fang Zheng, Yu-Qi Feng, Bi-Feng Yuan

**Affiliations:** Key Laboratory of Analytical Chemistry for Biology and Medicine (Ministry of education), Department of Chemistry, Wuhan University, Wuhan, 430072 China; Center for Gene Diagnosis, Zhongnan Hospital of Wuhan University, Wuhan, 430071 China

**Keywords:** 5-methylcytosine, 5-hydroxymethylcytosine, Aging, Hydrophilic-interaction liquid chromatography, Mass spectrometry

## Abstract

**Background:**

Aging is a complex phenomenon and characterized by a progressive decline in physiology and function of adult tissues. However, it hasn’t been well established of the correlation between aging and global DNA methylation and hydroxymethylation that regulate the growth and development of higher organisms.

**Results:**

We developed an on-line trapping/capillary hydrophilic-interaction liquid chromatography/electrospray ionization-mass spectrometry method for ultra-sensitive and simultaneous quantification of 5-methylcytosine (5-mC) and 5-hydroxymethylcytosine (5-hmC) in genomic DNA from human blood. Limits of detection for 5-mC and 5-hmC were 0.04 and 0.13 fmol, respectively. The imprecision and recovery of the method were determined with the relative standard deviations (RSDs) and relative errors being <11.2 and 14.0 %, respectively. We analyzed the contents of 5-mC and 5-hmC in genomic DNA of blood from 238 healthy people aged from 1 to 82 years. The results showed that 5-hmC content was significantly decreased and highly correlated with aging process, while 5-mC only showed slight correlation with age. We then established a DNA hydroxymethylation age model according to 5-hmC content with a mean absolute deviation (MAD) of approximate 8.9 years. We also calculated the mean relative error (MRE) using the predicted ages based on the age model and the chronological ages. The results showed that the MRE was 18.3 % for samples with ages from 20 to 82 years (95 % confidence interval, *N* = 190).

**Conclusions:**

The global DNA hydroxymethylation represents a strong and reproducible mark of chronological age, which could be potentially applied in health assessment and prevention of diseases. The identification of biological or environmental factors that influence DNA hydroxymethylation aging rate may permit quantitative assessments of their impacts on health.

**Electronic supplementary material:**

The online version of this article (doi:10.1186/s13148-015-0109-x) contains supplementary material, which is available to authorized users.

## Background

Aging is a complex phenomenon and characterized by a progressive decline in the ability to deal with physiological challenges over time [1]. It is known that mutations in genes can severely influence aging process. However, not all events seen in aging can be explained by the information carried by DNA sequence. Epigenetic marks, which control gene expression through changes beyond DNA sequence, show involvement in aging process.

It has been known for 40 years that aging is linked to the change of DNA cytosine methylation (5-methylcytosine [5-mC]) [2], which is one of the best-characterized epigenetic modifications associated with various physiological and pathological processes [3, 4]. Although there has been a great progress, the role of epigenetic changes in aging process is still a mystery [5]. Both DNA hypermethylation and hypomethylation have been observed during aging by various studies that characterize DNA methylation in site-specific CpGs sites [6–12]. The controversial results could be attributed to the difference of the selected CpGs sites in certain genomic regions. In addition, there seems to be tissue-specific differences in the age-related DNA methylation profiles [13, 14].

In recent years, 5-hydroxymethylcytosine (5-hmC) has been discovered in mammals and viewed to be “the sixth base” in addition to adenine, cytosine, thymine, guanine, and 5-mC [15]. It has been proposed that, similar to 5-mC, 5-hmC may also function as an epigenetic marks by itself [16]. Mapping of 5-hmC in tissues and cell lines demonstrated that the genomic distribution of 5-hmC is nonrandom and distinct from that of 5-mC [17, 18], with 5-hmC being especially enriched in the gene bodies and enhancers [19], indicating that 5-hmC may play important roles on cellular differentiation and epigenetic regulation [20, 16]. In addition, some studies including our recent report demonstrated that 5-hmC significantly decreased in various tumors, suggesting that 5-hmC is also associated with tumor formation and development [21–26].

5-hmC content has been shown to alter in mouse aging brain [27, 28]. Age-associated changes in global DNA hydroxymethylation in human are not yet known. Like 5-mC, change in 5-hmC may occur during aging, potentially leading to downstream changes in transcription and cellular physiological functions. In this study, we attempt to determine the impact of aging on global DNA methylation and hydroxymethylation. Generally, 5-hmC contents in cultured cells and blood cells are approximate one order of magnitude lower compared with tissue samples [29]. In this respect, we developed an online trapping/*c*HILIC/ESI-MS system for ultra-sensitive and simultaneous quantification of 5-mC and 5-hmC in blood genomic DNA. We prepared hydrophilic organic-silica hybrid monolith using the sol–gel combined with “thiol-ene” click reaction for the separation of nucleosides. A poly(MAA-*co*-EGDMA) monolithic capillary was used as the on-line trapping column. Using this on-line trapping/*c*HILIC/ESI-MS analytical system, 5-mC and 5-hmC contents in genomic DNA of human blood from 238 healthy people aged from 1 to 82 years were determined. Our results showed that 5-hmC content was significantly decreased and negatively correlated with aging, suggesting 5-hmC may have the potential to substantially contribute to aging phenotypes.

## Results

### Development of on-line trapping/*c*HILIC/ESI-MS analytical platform

Recently, our group successfully developed a novel method for one-pot preparation of organic-silica hybrid capillary monolithic column by sol–gel combined with “thiol-ene” click reaction, which allows higher yield and less by-products compared to other methods [30–32]. Using the “thiol-ene” click reaction, here we successfully prepared hydrophilic organic-silica hybrid monolith. The formed monolith was homogeneous and attached well to the inner wall of the capillary (Additional file 1: Figure S1A), which can provide effective mass transfer and high stability. The specific surface area of the capillary monolithic column was 302 m^2^/g with 4.5 nm mesoporous distribution examined by nitrogen adsorption-desorption experiments (Additional file 1: Figure S1B). Compared with the polymer monolith that we prepared previously [21], the organic-silica hybrid monolith exhibited much better stability and permeability as well as larger specific surface area. The prepared hydrophilic organic-silica hybrid monolith also demonstrated good chromatographic performance for the analysis of nucleosides (Additional file 1: Figure S2A).

**Fig. 1:**
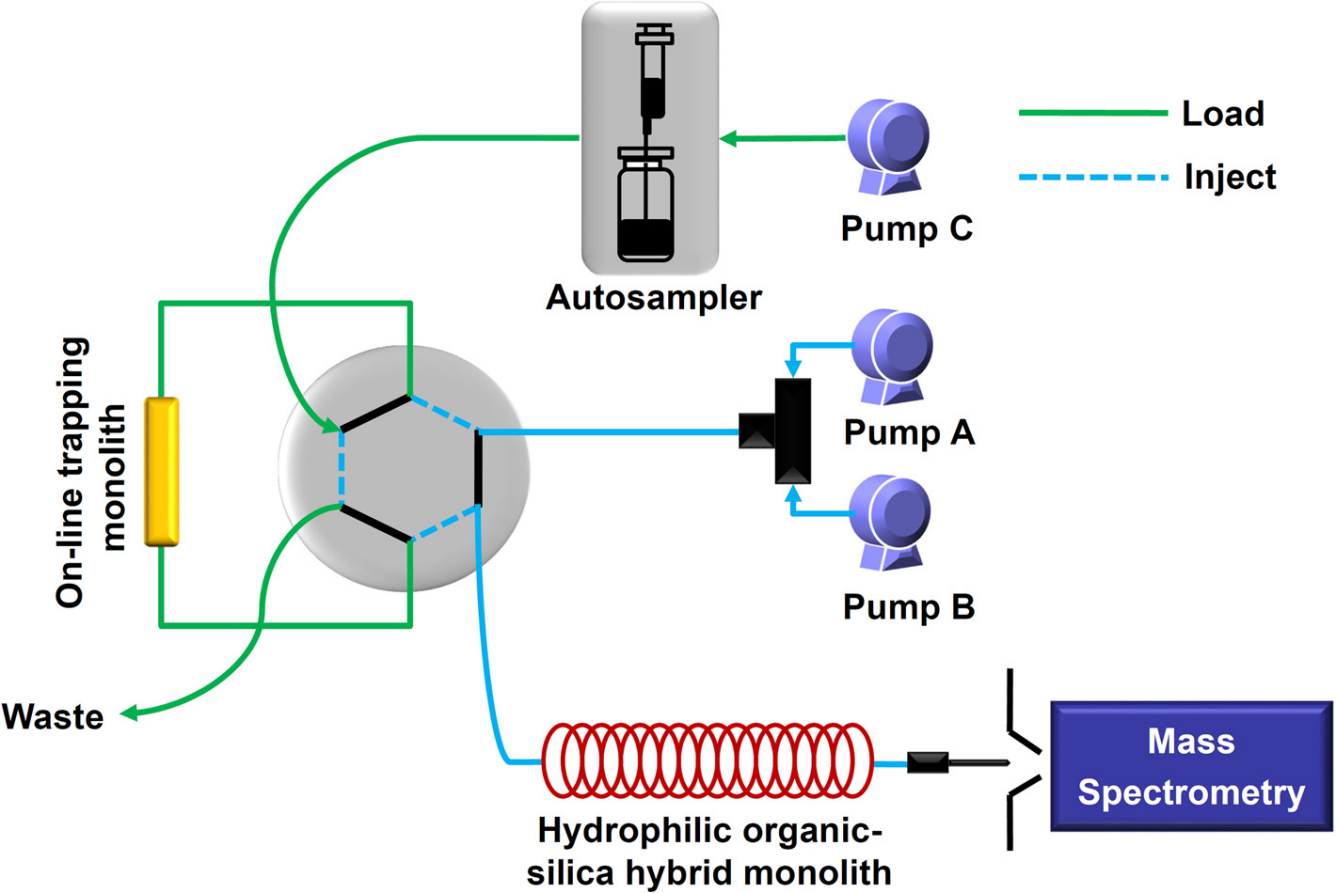
Experimental setup for the analysis of nucleosides (5-hmdC, 5-mdC, dC, dG, dA, T, C, G, A, and U) by on-line trapping/*c*HILIC/ESI-MS

The schematic diagram for the on-line trapping/*c*HILIC/ESI-MS system was shown in Fig. 1. The on-line trapping approach was established using hydrophobic poly(MAA-*co*-EGDMA) monolith, and the conditions were optimized to achieve the best analytical performance for the detection of 5-mC and 5-hmC. Firstly, we optimized the flow rate of the loading solution. When the loading flow rate increased from 2 to 20 μL/min, the peak areas of 5-mdC and 5-hmdC didn’t changed apparently (Additional file 1: Figure S3A). When the loading flow rate increased from 20 to 40 μL/min, the peak areas of the two analytes dropped. Therefore, we used 15 μL/min as the loading flow rate.

Secondly, we optimized the washing volume. The results showed that when the washing volume was more than 4 μL, the peak areas of 5-mdC dropped dramatically (Additional file 1: Figure S3B). Therefore, we chose the 4 μL as the washing volume. And then we optimized the desorption volume. When the desorption volume increased from 400 to 1500 nL, the signal-to-noise (S/N) ratio of 5-mdC and 5-hmdC increased (Additional file 1: Figure S3C). However, further increase of the desorption volume from 1500 to 2500 μL caused the decrease of the signal-to-noise (S/N) ratio of 5-mdC and 5-hmdC (Additional file 1: Figure S3C), which can be attributed to the extending of the sample zone with the further increased desorption volume. As a result, we chose 1500 nL as the desorption volume. Good linearities were obtained with linear coefficient *R*^2^ > 0.9953 in the range from 0 to 400 pmol of 5-mdC and 5-hmdC (Additional file 1: Figure S3D), demonstrating that the poly(MAA-*co*-EGDMA) on-line trapping column possessed high loading capacity.

Taken together, the optimized on-line trapping conditions consisted of a loading flow rate of 15 μL/min, a washing volume of 4 μL, and a desorption volume of 1.5 μL. Under the optimized conditions, the nucleosides can be well enriched with the recoveries of 10 nucleosides being between 83.9 and 99.2 % (Data not shown).

Here, we employed in-source collision-activated dissociation (CAD) for the identification and quantification of nucleosides according to our previously described method [21]. With this strategy, the generation of [M + Na]^+^ and [M + K]^+^ ions was significantly minimized, which therefore increased the detection sensitivities of 5-mdC and 5-hmdC for more than one order of magnitude [21].

### Method validation

The calibration curve of 5-mdC and 5-hmdC was constructed by plotting the mean peak area ratio of 5-mdC/dC or 5-hmdC/dC versus the mean molar ratio of 5-mdC/dC or 5-hmdC/dC based on the data obtained from triplicate measurements. The results showed that good linearities within the range of 0.1–10 % of 5-mdC/dC and 0.002–0.1 % of 5-hmdC/dC were obtained with coefficient values (*R*^2^) being great than 0.9951 (Table 1). The limit of detection (LODs) and the limit of quantification (LOQs), defined as the amounts of the analytes at a signal-to-noise ratio (S/N) of 3 and 10, respectively, were 0.04 and 0.13 fmol for 5-mdC and 0.19 and 0.62 fmol for 5-hmdC (Table 1), which were better than the LODs obtained the most sensitive detection methods previously established [29, 33, 21, 34] (Additional file 1: Table S1). The high sensitivity of this on-line trapping/*c*HILIC/ESI-MS method could be attributed to the employment of in-source CAD, on-line trapping approach, and the miniaturized hydrophilic separation column.

**Table 1:** Linearities, LOQs, and LODs for 5-mdC and 5-hmdC obtained by on-line trapping/*c*HILIC/ESI-MS

Analytes	Linear range (vs.[dC],%)	Regression line		*R* ^2^	LOD (fmol)	LOQ (fmol)
		Slope	Intercept			
5-mdC	0.1–10	0.0193 ± 0.0007	−0.0017 ± 0.0003	0.9951	0.04	0.13
5-hmdC	0.002–0.1	0.0126 ± 0.0002	0.0001 ± 0.00001	0.9997	0.19	0.62

We further validated the method with the synthesized 5-mC- or 5-hmC-containing oligodeoxynucleotide by comparing the measured 5-mdC or 5-hmdC contents to the theoretical 5-mdC or 5-hmdC content (Additional file 1: Table S2). The results showed that good accuracy could be achieved, which are manifested by relative errors being −9.2–14.0 % (Additional file 1: Table S3).

The reproducibility of the method was evaluated by the measurement of intra- and inter-day imprecisions (Additional file 1: Table S4). The intra- and inter-day RSDs were calculated with different amounts of 5-mdC and 5-hmdC spiked in nucleosides mixture. Three parallel measurements over a day gave the intra-day RSDs, and the inter-day RSDs were determined by measuring samples five times independently for three consecutive days. The results showed that the intra- and inter-day RSDs for 5-mdC and 5-hmdC were less than 10.4 and 13.2 %, respectively (Additional file 1: Table S4). The result indicated that the on-line trapping/*c*HILIC/ESI-MS method was reliable for the simultaneous quantification of 5-mC and 5-hmC in genomic DNA.

### Measurement of 5-mC and 5-hmC in genomic DNA from human blood

Using the established on-line trapping/*c*HILIC/ESI-MS platform, we further quantified 5-mC and 5-hmC contents in genomic DNA of human blood.

Additional file 1: Figure S2B shows the extracted-ion chromatogram of the hydrolysis product of human blood genomic DNA. The chromatograms of 5-mdC and 5-hmdC were extracted at *m/z* 126.068 (0.01) and 142.062 (0.01), respectively. Due to the high sensitivity of the developed method, we can easily quantified both 5-mdC and 5-hmdC using only 2 ng genomic DNA (Additional file 1: Figure S2B).

We also compared 5-mdC and 5-hmdC levels measured by our developed on-line trapping/*c*HILIC/ESI-MS method with normal LC/MS method (Additional file 1). The results showed that 5-mdC and 5-hmdC contents in the examined 18 human blood samples were comparable using these two methods, with relative errors being −12.4 to 15.7 % (Additional file 1: Table S5), indicating that the on-line trapping/*c*HILIC/ESI-MS method is reliable for the determination of 5-mC and 5-hmC in genomic DNA. However, due to the detection, sensitivities of 5-mC and 5-hmC by normal LC/MS method were much lower compared to on-line trapping/*c*HILIC/ESI-MS method, therefore, approximate 2 μg genomic DNA is required for the accurate quantification of 5-mC and 5-hmC by normal LC/MS method.

### Correlation analysis of 5-mC and 5-hmC contents with age

**Fig. 2:**
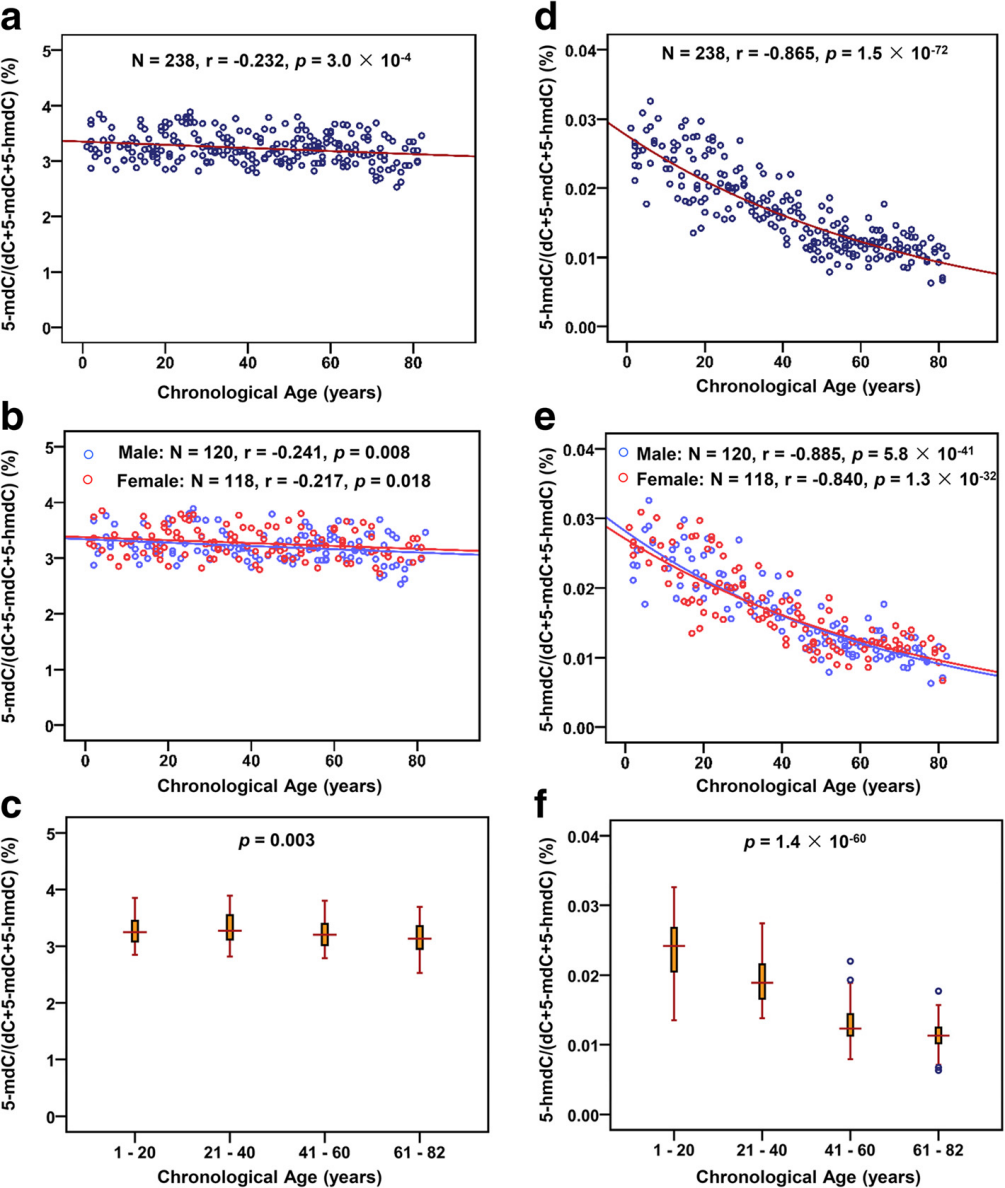
Correlation analysis of DNA methylation and hydroxymethylation with age. (**a**) Exponential regression of 5-mC content in genomic DNA of blood with age. (**b**) Exponential regression of 5-mC content in genomic DNA of blood with age using different genders. (**c**) Comparison of 5-mC content at different age stages. (**d**) Exponential regression of 5-hmC content in genomic DNA of blood with age. The equation of the exponential regression is “y = 0.0276e^-0.0135x^”, where y represents 5-hmC content in genomic DNA and x represents chronological age. (**e**) Exponential regression of 5-hmC content in genomic DNA of blood with age using different genders. (**f**) Comparison of 5-hmC content at different age stages. Each point represents the 5-mC or 5-hmC content in one sample

The contents of 5-mC and 5-hmC in the blood genomic DNA from 238 people aged from 1 to 82 years were determined by on-line trapping/*c*HILIC/ESI-MS (Additional file 1: Tables S6). To evaluate the effect of aging on DNA methylation and hydroxymethylation, we performed linear and exponential regression to establish the correlation between 5-mC and 5-hmC contents in genomic DNA and chronological age. The results showed slight correlation between 5-mC and chronological age (linear regression—*r* = −0.230, *p* = 3.5 × 10^−4^; exponential regression—*r* = −0.232, *p* = 3.0 × 10^−4^, *N* = 238). Figure 2a shows the exponential regression of the correlation between 5-mC and chronological age. And no gender difference was observed of the correlation between 5-mC content and age (Fig. 2b). We also performed the comparison of DNA methylation during different age stages with one-way ANOVA analysis, and the results showed that 5-mC contents slightly decreased in aged samples (one-way ANOVA, *p* = 0.003, Fig. 2c).

Different with 5-mC, 5-hmC content was significantly decreased and highly correlated with aging (linear regression—*r* = −0.857, *p* = 4.3 × 10^−70^; exponential regression—*r* = −0.865, *p* = 1.5 × 10^−72^, *N* = 238). The linear regression of the correlation between 5-hmC and chronological age is shown in Additional file 1: Figure S4. Figure 2d shows the exponential regression of the correlation between 5-hmC and chronological age. And the correlation between 5-hmC content and age was regardless of gender difference (Fig. 2e). In addition, 5-hmC contents at different age stages also exhibited decreased tendency. Compared with young samples, DNA hydroxymethylation levels significantly decreased in aged samples (one-way ANOVA, *p* = 1.4 × 10^−60^) (Fig. 2f).

Since dietary habit may play certain roles on the contents change of 5-mC and 5-hmC with aging, here, we also analyzed other 172 blood samples with ages ranging from 3 to 88 years from Henan province, China. Similarly, we performed linear and exponential regression to establish the correlation between 5-mC and 5-hmC contents in genomic DNA and chronological age. As shown in Additional file 1: Figure S5A, there is slight correlation between 5-mC and chronological age (linear regression—*r* = −0.482, *p* = 2.6 × 10^−11^; exponential regression—*r* = −0.489, *p* = 1.2 × 10^−11^, *N* = 172). No gender difference was observed of the correlation between 5-mC content and age (Additional file 1: Figure S5B), and 5-mC contents slightly decreased in aged samples (one-way ANOVA, *p* = 7.3 × 10^−10^, Additional file 1: Figure S5C). As for 5-hmC, a significant decrease highly correlated with aging was observed (linear regression—*r* = −0.887, *p* = 1.4 × 10^−58^; exponential regression—*r* = −0.908, *p* = 8.5 × 10^−66^, *N* = 172) without gender difference (Additional file 1: Figure S5D, E, F). These results are consistent with the results obtained by using the samples collected from Zhongnan Hospital of Wuhan University (Hubei, China), indicating that the decline of 5-hmC with aging is regardless of dietary habit and regions.

### DNA hydroxymethylation predictor of age

**Fig. 3:**
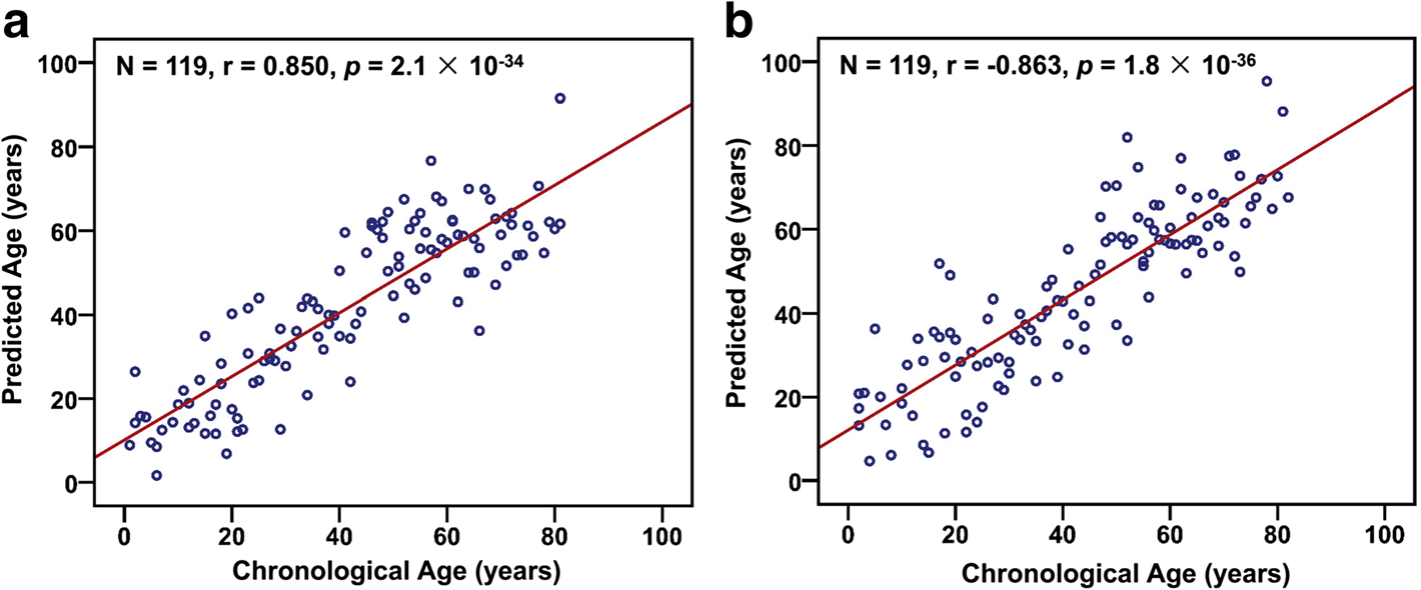
Chronological age (*x*-axis) versus DNA hydroxymethylation age in the training group (**a**) and test group (**b**)

We further built a DNA hydroxymethylation age model to predict age. In this respect, we randomly divided the data into two independent dataset, namely training and test groups. To estimate the accuracy of the aging model, the mean absolute deviation (MAD) between predicted and chronological age was calculated using exponential regression based on the training data. DNA hydroxymethylation age model was constructed using 5-hmC content in training dataset by exponential regression (*r* = 0.850, *p* = 2.1 × 10^−34^, MAD = 8.7 years, *N* = 119) (Fig. 3a). Then the accuracy of the model was validated by the test dataset, and the result showed that the model fit well in the test group (*r* = 0.863, *p* = 1.8 × 10^−36^, MAD = 9.3 years, *N* = 119) (Fig. 3b). Similarly, as for the samples collected from Henan province, DNA hydroxymethylation age model was also built from training data by exponential regression (*r* = 0.896, *p* = 2.4 × 10^−31^, MAD = 8.4 years, *N* = 86) (Additional file 1: Figure S6A) and validated by the test dataset (*r* = 0.928, *p* = 1.2 × 10^−37^, MAD = 7.4 years, *N* = 86) (Additional file 1: Figure S6B).

**Fig. 4:**
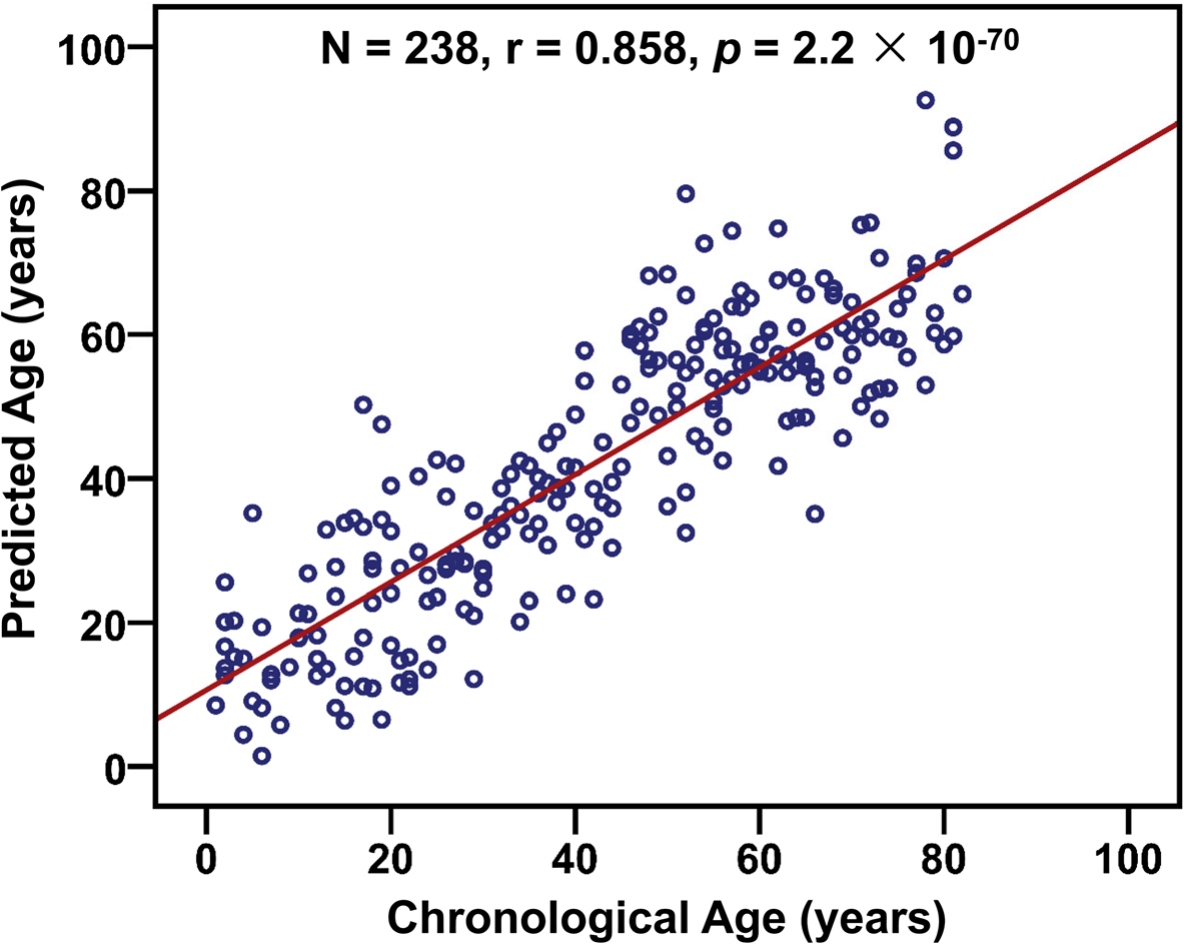
Predicted age versus chronological age of all subjects using a leave-one-out model. A multivariate regression model was fit on all but one sample and its predicted age (*y*-axis) was related to the chronological age of the left out sample (*x*-axis)

To provide an unbiased estimate of predictive accuracy for age, we further used a leave-one-out analysis where the multivariate regression model was fit on all but one subject and its prediction was related to the chronological age of the left-out subject. The predicted values are highly correlated with the chronological age in all samples (*r* = 0.858, *p* = 2.2 × 10^−70^, *N* = 238), and the MAD between the predicted and chronological age is 8.9 years (Fig. 4), which is similar as that obtained by exponential regression analysis (Fig. 3). And similar results were obtained using the samples collected from Henan province (*r* = 0.910, *p* = 2.3 × 10^−66^, MAD = 7.8 years, *N* = 172, Additional file 1: Figure S7).

We also calculated the mean relative error (MRE) using the predicted ages based on the age model and the chronological ages. The results showed that the MRE was 18.3 % for samples with ages from 20 to 82 years old (95 % confidence interval, *N* = 190). With ages from 1 to 82 years old, the MRE was 53.7 %, which is relatively high. Since the relative errors were calculated based on the ratios of the calculated differences between predicted ages and chronological ages over chronological ages, the chronological ages of young people are relatively small, therefore, the calculated relative errors will be larger than older people, which cause relatively high MRE for young people. The results indicated that the built model is more accurate at older ages.

## Discussion

Cell state is maintained by epigenetic memory, which in part reflects the developmental history of a cell [35]. Epigenetic alterations that may change the function of the cells and organisms could have meaningful downstream effects. Early studies suggested that global DNA methylation may increase in rat kidneys [2] or decrease in the brain, liver, and small intestine mucosa tissues of mice [36]. And recent studies indicated that DNA methylation in certain CpG sites has shown to be relative to chronological age [8, 11]. The controversial results of these previous studies as well as the less exploration of the effect of human aging on DNA epigenetic modifications encourage us to comprehensively investigate the association between the global 5-mC content and its oxidation product of 5-hmC content in genomic DNA and age.

To this end, in this study, we developed a highly sensitive on-line trapping/*c*HILIC/ESI-MS method to precisely and simultaneously measure global DNA methylation and hydroxymethylation, which can be achieved using only 2 ng of genomic DNA. The detection limits were better than the most sensitive detection methods previously established. Using this analytical platform, we reported the alteration of global DNA methylation and hydroxymethylation in genomic DNA of human blood during aging process. The results suggested that 5-hmC content was significantly decreased and highly correlated with aging process (*N* = 238, *r* = −0.865, *p* = 1.5 × 10^−72^), while 5-mC only slightly decreased and exhibited weak correlation with aging (*N* = 238, *r* = −0.232, *p* = 3.0 × 10^−4^).

Several studies have investigated the genomic methylation maps of CpG islands in individuals of increasing age. Bocklandt et al. [37] identifies 88 sites in 80 genes for which the degree of DNA cytosine methylation is significantly correlated with age. Furthermore, they validated CpG sites in the promoters of three genes and built a regression model to predict the age of individual with an average accuracy of 5.2 years. Weidner et al. [38] performed a comprehensive analysis of methylation profiles to narrow down 102 age-related CpG sites in blood. They further built an age prediction using three age-related CpG sites by bisulfite pyrosequencing of 151 blood samples with a MAD from chronological age of 5 years. The accuracy of these age models based on specific CpG sites was slightly better than the DNA hydroxymethylation age model we developed in the current study. However, these methods require extensive sequencing and then the age-related CpG sites were identified based on statistical analysis. The practical application still relies on bisulfite sequencing or next generation sequencing, which is time-consuming and costly. With our developed on-line trapping/*c*HILIC/ESI-MS method, the quantification of global DNA methylation and hydroxymethylation is simple, cost-effective and can be finished within 25 min. The mechanism that drives the significant decrease of DNA hydroxymethylation during aging is still unknown. Here, we investigated the correlation of blood cells composition with the contents of 5-mC and 5-hmC (Additional file 1: Table S7). And there are very weak correlations of blood cells composition with the contents of 5-mC and 5-hmC (Additional file 1: Table S8). The results indicated that cells composition may not play a role on the contents change of 5-hmC during aging.

Intrinsic (genetically determined) as well as extrinsic (environmentally induced) factors could influence the aging process through epigenetic mechanisms. Three factors may attribute to the decreased hydroxymethylation with aging. Firstly, it is possible that environmental exposure over time will activate cellular programs associated with consistent and predictable changes in the epigenome. For example, stress has been shown to alter gene expression patterns through specific changes in DNA methylation [39]. Secondly, 5-hmC can be lost passively through cell divisions besides active removal of 5-hmC through the oxidation to 5-formylcytosine and 5-carboxylcytosine [40]. During aging, cytosine in the CpG sites may not be efficiently methylated by DNA methyltransferases and then oxidized by TET proteins to 5-hmC upon cell divisions, since the parent strand of DNA with half hydroxymethylated cytosine is not an appropriate substrate [41]. Therefore, the increase of cellular divisions may be one of the causes responsible for the decrease in DNA hydroxymethylation shown in the blood samples during aging. Thirdly, the slight decline of 5-mC during aging may also contribute to the decreased 5-hmC since 5-hmC is mainly generated through the oxidation of 5-mC by TET proteins. These mechanisms lead to differences between DNA hydroxymethylation of aging individuals, suggesting that quantitative measurements of hydroxymethylation states may identify factors involved with slowed or accelerated rates of aging. And further investigation is required to elucidate the detailed mechanism of 5-hmC decrease during aging.

## Conclusions

In this study, we developed a highly sensitive on-line trapping/*c*HILIC/ESI-MS method for ultra-sensitive and simultaneous quantification of 5-mC and 5-hmC in genomic DNA from human blood. Using this analytical platform, we reported the alteration of global DNA methylation and hydroxymethylation in genomic DNA of human blood during aging process. The results showed that 5-hmC content was significantly decreased and highly correlated with aging process, while 5-mC only showed slight correlation with age. The analysis of global 5-hmC in genomic DNA only requires the enzymatic digestion of DNA followed by subsequent content analysis, which is much easier and more cost-effective than the site-specific evaluation of DNA methylation. Therefore, it is conceivable that with the improvement of DNA hydroxymethylation age model, biological age as measured from molecular profiles might one day be potentially applied in health assessment and prevention of diseases.

## Methods

### Chemicals and reagents

2′-deoxycytidine (dC), 2′-deoxyguanosine (dG), 2′-deoxyadenosine (dA), thymidine (T), and 5-methyl-2′-deoxycytidine (5-mdC) were purchased from Sigma-Aldrich (Beijing, China), and 5-hydroxymethyl-2′-deoxycytidine (5-hmdC) was purchased from Berry & Associates (Dexter, MI). Chromatographic grade methanol was purchased from Merck (Darmstadt, Germany). Chromatographic grade isopropanol, acetonitrile (ACN), and formic acid were from Tedia Co. Inc. (Fairfield, OH). All other solvents and chemicals used were of analytical grade. S1 nuclease and alkaline phosphatase were from Takara Biotechnology Co., Ltd (Dalian, China). Phosphodiesterase I was purchased from Sigma-Aldrich (St. Louis, MO). Water used throughout all experiments was purified by a Milli-Q water purification apparatus (Millipore, Bedford, MA).

Fused-silica capillary (50 μm i.d. × 360 μm o.d. and 75 μm i.d. × 360 μm o.d.) was purchased from Yongnian Optic Fiber Plant (Hebei, China). Tetramethoxysilane (TMOS), 3-mercaptopropyltrimethoxysilane (MPTMS), and 3-(triethoxysilyl)propyl methacrylate were purchased from Wuhan University Silicone New Material (Wuhan, China). Azobisisobutyronitrile (AIBN), poly(ethylene glycol)-6000 (PEG-6000), toluene, dodecanol, and *N,N*-dimethylformamide (DMF) were all purchased from Shanghai Chemical Reagent Corporation (Shanghai, China). AIBN was purified by recrystallization from ethanol at 40 °C. *N*-acryloyl-tris-(hydroxymethyl)aminomethane was purchased from Sigma-Aldrich (Beijing, China). Methacrylic acid (MAA, 95 wt%) and ethylene glycol dimethacrylate (EGDMA, 95 wt%) were purchased from Acros Organics (Morris Plains, NJ).

### Human blood samples

The study was approved by medical ethic committee of Zhongnan Hospital of Wuhan University and met the declaration of Helsinki. The research consisted of healthy individuals that randomly selected after medical check-up in the physical examination center of Zhongnan Hospital of Wuhan University (Hubei, China) and the First Affiliated Hospital of Zhengzhou University (Henan, China) between July of 2014 and June of 2015. Persons who have history of tumor, cardiovascular disease, diabetes, hypertension, metabolic syndrome, endocrine system diseases, liver, and kidney system diseases were excluded. The informed consents were obtained from all subjects or their guardians.

### DNA extraction and enzymatic digestion

Blood genomic DNA was isolated using the E.Z.N.A.™ Blood DNA Kit (Omega Bio-Tek Inc., Norcross, GA) according to the manufacture’s recommended protocol. The concentration of the purified DNA was determined using B-500 spectrophotometer (Metash Instruments Co., Ltd., Shanghai, China). The enzymatic digestion of genomic DNA was performed according to the previously described method with slight modification [21]. Briefly, genomic DNA in 8.5 μL H_2_O was denatured by heating at 95 °C for 5 min and then chilled on ice for 2 min. After adding 1 μL of S1 nuclease buffer (30 mmol/L CH_3_COONa, pH 4.6, 280 mmol/L NaCl, 1 mmol/L ZnSO_4_) and 100 units (0.5 μL) of S1 nuclease, the mixture was incubated at 37 °C for 4 h. Then 24.5 μL H_2_O, 4 μL alkaline phosphatase buffer (50 mM Tris–HCl, 10 mM MgCl_2_, pH 9.0), 0.001 units (1 μL) of venom phosphodiesterase, and 15 units (0.5 μL) of alkaline phosphatase were subsequently added. The mixture was incubated at 37 °C for an additional 2 h. The resulting solution was extracted with phenol/chloroform (1/1, *v/v*) once and chloroform twice. The extracted nucleosides were lyophilized to dryness and then reconstituted in 200 μL of ACN/H_2_O (99/1, *v/v*). The obtained solution was centrifugal at 12,000 rpm for 5 min to remove salts. Then the supernatant was used for subsequent 5-mdC and 5-hmdC analysis by on-line trapping/*c*HILIC/ESI-MS.

### Preparation of monolithic capillary columns

For the preparation of hydrophilic organic-silica hybrid monolith, a polymerization mixture containing acetic acid (0.01 mol/L, 500 mg), poly(ethylene glycol)-6000 (45 mg), tetramethoxysilane (185 mg), 3-mercaptopropyltrimethoxysilane (15 mg), *N*-acryloyl-tris-(hydroxymethyl)aminomethane (15 mg), and azobisisobutyronitrile (1 mg) was completely mixed and degassed by ultra-sonication for 5 min. The mixture was then manually introduced into the activated fused silica capillary (75 μm i.d. × 360 μm o.d.) by a syringe. After both ends of the capillary were sealed with two pieces of silicone rubber, the mixture was incubated at 40 °C for 12 h for simultaneous polymerization and “thiol-ene” click reaction. The resulting monolith was completely flushed with water and ACN sequentially to remove the poly(ethylene glycol)-6000 and other residuals. The schematic diagram for the preparation of hydrophilic organic-silica hybrid monolith was shown in Additional file 1: Figure S8.

The on-line trapping monolithic capillary column of poly(MAA-*co*-EGDMA) was prepared according to our previously described method [42]. To activate the silanol groups, the fused-silica capillaries were sequentially washed with 1 mol/L NaOH for 2 h, H_2_O for 30 min, 1 mol/L HCl for 1 h, H_2_O for 30 min, and methanol for 30 min followed by drying under nitrogen flow at 160 °C for 6 h. The inner face of activated fused-silica capillaries were derivatized with 3-(triethoxysilyl)propyl methacrylate and then dried with the nitrogen gas. Then, a polymerization mixture containing MAA (48 mg), EGDMA (420 mg), toluene (110 mg), dodecanol (869 mg), and AIBN (4.5 mg) was completely mixed and degassed by ultra-sonication for 5 min. The mixture was then manually introduced into the 3-(triethoxysilyl)propyl methacrylate derivatized silica capillary (50 μm i.d. × 360 μm o.d.) by a syringe. After both ends of the capillary were sealed with two pieces of silicone rubber, the mixture was incubated at 60 °C for 12 h. The resulting monolith was completely flushed with water and ACN sequentially to remove the residuals.

### Characterization of hydrophilic organic-silica hybrid monolith

The specific surface area of prepared hydrophilic organic-silica hybrid monolithic materials was measured by nitrogen adsorption-desorption experiments using a JW-BK-specific surface area and pore size analyzer (JWGB Sci & Tech Co., Ltd., Beijing, China). Before measurement, the monolithic cubic pieces were evacuated in vacuum and heated to 120 °C for 4 h to remove the physically adsorbed substances. Specific surface area values were determined by the Brunauer-Emmett-Teller (BET) equation at P/P_0_ between 0.05 and 0.3. The microscopic morphology of the monoliths was examined by scanning electron microscopy (SEM) using a Quanta 200 scanning electron microscope (FEI Company, Holland).

### On-line trapping/*c*HILIC/ESI-MS

The on-line trapping/*c*HILIC/ESI-MS system consisted of a micrOTOF-Q orthogonal-accelerated TOF mass spectrometer (Bruker Daltonics, Bremen, Germany) with an ESI source (Turbo Ionspray) and two Shimadzu LC-20 AD nano pump (Tokyo, Japan), a Shimadzu LC-20 AD pump (Tokyo, Japan), a FVC nano valve of two positions (Tokyo, Japan), a SIL-20 AC auto sampler, and a DGU-20A3 degasser. A 0.5-cm-long poly(MAA-*co*-EGDMA) monolith (50 μm i.d. × 360 μm o.d.) was employed as the on-line trapping column. The hydrophilic organic-silica hybrid monolith (30-cm long, 75 μm i.d. × 360 μm o.d.) was used as analytical column, and the flow rate was set at 1 μL/min. The hydrophilic organic-silica hybrid monolith was connected to a PicoTip™ (New Objective) nano-spray tip (360 μm outer diameter, 10 μm inner diameter) with a zero-dead-volume union (Upchurch Scientific) to minimize postcolumn dead volume. Formic acid in water (0.01 %, *v/v*, solvent A) and formic acid in ACN (0.01 %, *v/v*, solvent B) was employed as mobile phase. An isocratic elution of 96 % B was employed.

Data acquisition and processing were performed using Bruker Daltonics Control 3.4 and Bruker Daltonics Data analysis 4.0 software. The mixture of the 5-mdC and 5-hmdC standards sample was employed to optimize the mass spectrometry conditions under positive ion mode. The optimized mass spectrometry parameters for the analysis were as follows: capillary voltage −1.5 kV; dry gas 4.0 L/min; dry temperature 180 °C; funnel 1 RF 200.0 Vpp; funnel 2 RF 200.0 Vpp; ISCID energy 5.0 eV; hexapole RF 200.0 Vpp; quadrupole ion energy, 15 eV; collision energy, 12 eV; collision RF, 150 Vpp; and pre pulse storage 6.0 μs. Spectra were acquired by summarizing 5000 single spectra in the *m/z* range of 100 to 400. Full scan mode was used. The identification and quantification ions for nucleosides were listed in Additional file 1: Table S9.

The genome-wide contents of 5-mC and 5-hmC measured by on-line trapping/*c*HILIC/ESI-MS were calculated using the following formula:

$$ 5\hbox{-} \mathrm{m}\mathrm{C},\kern0.5em \%\left(\mathrm{or}\kern0.5em 5\hbox{-} \mathrm{h}\mathrm{m}\mathrm{C},\kern0.5em \%\right)=\frac{M_{5\hbox{-} \mathrm{m}\mathrm{d}\mathrm{C}}\left(\mathrm{or}\kern0.5em {M}_{5\hbox{-} \mathrm{hm}\mathrm{d}\mathrm{C}}\right)}{M_{\mathrm{dC}}+{M}_{5\hbox{-} \mathrm{m}\mathrm{d}\mathrm{C}}+{M}_{5\hbox{-} \mathrm{hm}\mathrm{d}\mathrm{C}}}\times 100\ \% $$

where *M*_5-mdC_, *M*_5-hmdC_, and *M*_dC_ are the molar quantities of 5-mdC, 5-hmdC, and dC determined in DNA samples.

### Analysis of 5-mdC and 5-hmdC by normal LC/MS

Analysis of 5-mdC and 5-hmdC was performed on the LC/MS system consisting of an AB 3200 QTRAP mass spectrometer (Applied Biosystems, Foster City, CA) with an electrospray ionization source (Turbo Ionspray) and a Shimadzu LC-20 AD HPLC (Tokyo, Japan) with two LC-20 AD pumps, a SIL-20A autosampler, a CTO-20 AC thermostatted column compartment, and a DGU-20A3 degasser. Data acquisition and processing were performed using AB SCIEX Analyst 1.5 Software (Applied Biosystems, Foster City, CA). The analytical column was performed on a Shim-pack VP-ODS column (250 × 2.0 mm i.d., 5 μm, Shimadzu, Japan) with a flow rate of 0.2 mL/min at 35 °C. Five millimole per liter ammonium formate with 0.01 % formic acid in water (solvent A) and methanol (solvent B) was employed as mobile phase. A gradient of 0–5 min 5 % B, 5–10 min 5 to 30 % B, 10–15 min 30 to 50 % B, 15–25 min 50 % B, 25–26 min 50 to 5 % B, and 25–40 min 5 % B was used.

The mass spectrometry detection was performed under positive electrospray ionization mode. The target nucleosides were monitored by multiple reaction monitoring (MRM) using the mass transitions (precursor ions → product ions) of dA (252.4 → 136.2), dG (268.4 → 152.1), dC (228.4 → 112.2), T (243.3 → 127.2), rA (268.4 → 136.2), rG (284.5 → 152.2), rC (244.1 → 112.1), U (245.0 → 113.1), 5-mdC (242.3 → 126.1), and 5-hmdC (258.2 → 142.1). The MRM parameters of all analytes were optimized to achieve maximal detection sensitivity.

### Statistical analysis

All the statistical analyses were performed using SPSS 19.0. We estimated Spearman correlation coefficients for correlation study. And *p* < 0.05 were considered to be statistically significant. The predicted models of DNA methylation and hydroxymethylation aging model were made with a curve estimation analysis based on linear and exponential regression. To estimate the accuracy of the aging models, the mean absolute deviation (MAD) of predicted and chronological age was calculated using exponential regression based on the training data. Then we applied this age predictor model to the test group. To provide an unbiased estimate of predictive accuracy of age, we used a leave-one-out analysis where the regression model was fit on all but one subject and its prediction was related to the chronological age of the left-out subject.

## Additional file

Additional file 1:
**Supplementary Data Table S1.** Comparison of the developed on-line trapping/cHILIC/ESI-MS method with other methods. **Supplementary Data Table S2.** The preparation of the quality control (QC) samples with the synthesized 5-mC- and 5-hmC-containing oligodeoxynucleotides. **Supplementary Data Table S3.** Accuracy of the method for the detection of 5-mdC and 5-hmdC. **Supplementary Data Table S4.** Intra- and inter-day imprecision for the quantification of 5-mdC and 5-hmdC by on-line trapping/cHILIC/ESI-MS method. **Supplementary Data Table S5.** The comparison of on-line trapping/cHILIC/ESI-MS method with normal LC/MS method for the quantification of 5-mC and 5-hmC in genomic DNA from 18 blood samples. **Supplementary Data Table S6.** Measured contents of 5-mdC and 5-hmdC in genomic DNA of blood from 238 healthy persons (blood samples were collected from Zhongnan Hospital of Wuhan University, Hubei, China). **Supplementary Data Table S7.** Measured contents of 5-mdC and 5-hmdC in genomic DNA of blood samples from 172 healthy persons (blood samples were collected from the First Affiliated Hospital of Zhengzhou University, Henan, China). **Supplementary Data Table S8.** The Spearman correlation of the contents of 5-mC and 5-hmC with respect to blood cell composition. **Supplementary Data Table S9.** The qualitative and quantitative ions for the detection of nucleosides. **Supplementary Data Figure S1.** Characterizations of hydrophilic organic-silica hybrid monolith. **Supplementary Data Figure S2.** Extracted-ion chromatograms of nucleosides by on-line trapping/cHILIC/ESI-MS analysis. **Supplementary Data Figure S3.** Optimizations of the on-line trapping/cHILIC/ESI-MS conditions. **Supplementary Data Figure S4.** The linear regression of 5-hmC content in genomic DNA of blood with age. **Supplementary Data Figure S5.** Correlation analysis of DNA methylation and hydroxymethylation with age using blood samples from Henan province, China. **Supplementary Data Figure S6.** Chronological age (x-axis) versus DNA hydroxymethylation age. **Supplementary Data Figure S7.** Predicted age versus chronological age of all subjects using a leave-one-out model using samples from Henan province, China. **Supplementary Data Figure S8.** Schematic procedure for the preparation of hydrophilic organic-silica hybrid monolith by sol-gel method combined with ‘‘thiol-ene’’ click reaction in “one-pot”.

## Abbreviations

5-mC: 5-methylcytosine
5-hmC: 5-hydroxymethylcytosine
5-mdC: 5-methyl-2′-deoxycytidine
5-hmdC: 5-hydroxymethyl-2′-deoxycytidine
dC: 2′-deoxycytidine
dG: 2′-deoxyguanosine
dA: 2′-deoxyadenosine
*c*HILIC: capillary hydrophilic-interaction liquid chromatography
CAD: collision-activated dissociation
ESI: electrospray ionization
LOD: limit of detection
LOQ: limit of quantification
LC/MS: liquid chromatography/mass spectrometry
MRM: multiple reaction monitoring
qTOF–MS: quadrupole TOF–mass spectrometry
RE: relative error
RSD: relative standard deviation
S/N: signal-to-noise
TET: ten–eleven translocation
T: thymidine

## References

[1] Johnson FB, Sinclair DA, Guarente L (1999). Molecular biology of aging. Cell..

[2] Vanyushin BF, Nemirovsky LE, Klimenko VV, Vasiliev VK, Belozersky AN (1973). The 5-methylcytosine in DNA of rats. Tissue and age specificity and the changes induced by hydrocortisone and other agents. Gerontologia.

[3] Smith ZD, Meissner A (2013). DNA methylation: roles in mammalian development. Nat Rev Genet..

[4] Robertson KD (2005). DNA methylation and human disease. Nat Rev Genet..

[5] Gibbs WW (2014). Biomarkers and ageing: the clock-watcher. Nature..

[6] Richardson B (2003). Impact of aging on DNA methylation. Ageing Res Rev..

[7] Rakyan VK, Down TA, Maslau S, Andrew T, Yang TP, Beyan H (2010). Human aging-associated DNA hypermethylation occurs preferentially at bivalent chromatin domains. Genome Res..

[8] Horvath S (2013). DNA methylation age of human tissues and cell types. Genome Biol..

[9] Bork S, Pfister S, Witt H, Horn P, Korn B, Ho AD (2010). DNA methylation pattern changes upon long-term culture and aging of human mesenchymal stromal cells. Aging Cell..

[10] Heyn H, Li N, Ferreira HJ, Moran S, Pisano DG, Gomez A (2012). Distinct DNA methylomes of newborns and centenarians. Proc Natl Acad Sci U S A..

[11] Hannum G, Guinney J, Zhao L, Zhang L, Hughes G, Sadda S (2013). Genome-wide methylation profiles reveal quantitative views of human aging rates. Mol Cell..

[12] McClay JL, Aberg KA, Clark SL, Nerella S, Kumar G, Xie LY (2014). A methylome-wide study of aging using massively parallel sequencing of the methyl-CpG-enriched genomic fraction from blood in over 700 subjects. Hum Mol Genet..

[13] Thompson RF, Atzmon G, Gheorghe C, Liang HQ, Lowes C, Greally JM (2010). Tissue-specific dysregulation of DNA methylation in aging. Aging Cell..

[14] Maegawa S, Hinkal G, Kim HS, Shen L, Zhang L, Zhang J (2010). Widespread and tissue specific age-related DNA methylation changes in mice. Genome Res..

[15] Munzel M, Globisch D, Carell T (2011). 5-Hydroxymethylcytosine, the sixth base of the genome. Angew Chem Int Ed Engl..

[16] Branco MR, Ficz G, Reik W (2012). Uncovering the role of 5-hydroxymethylcytosine in the epigenome. Nat Rev Genet..

[17] Wu H, D'Alessio AC, Ito S, Wang Z, Cui K, Zhao K (2011). Genome-wide analysis of 5-hydroxymethylcytosine distribution reveals its dual function in transcriptional regulation in mouse embryonic stem cells. Genes Dev..

[18] Ficz G, Branco MR, Seisenberger S, Santos F, Krueger F, Hore TA (2011). Dynamic regulation of 5-hydroxymethylcytosine in mouse ES cells and during differentiation. Nature..

[19] Stroud H, Feng S, Morey Kinney S, Pradhan S, Jacobsen SE (2011). 5-Hydroxymethylcytosine is associated with enhancers and gene bodies in human embryonic stem cells. Genome Biol..

[20] Kriukiene E, Liutkeviciute Z, Klimasauskas S (2012). 5-Hydroxymethylcytosine—the elusive epigenetic mark in mammalian DNA. Chem Soc Rev..

[21] Chen ML, Shen F, Huang W, Qi JH, Wang Y, Feng YQ (2013). Quantification of 5-Methylcytosine and 5-Hydroxymethylcytosine in genomic DNA from hepatocellular carcinoma tissues by capillary hydrophilic-interaction liquid chromatography/quadrupole TOF mass spectrometry. Clin Chem..

[22] Yang H, Liu Y, Bai F, Zhang JY, Ma SH, Liu J (2013). Tumor development is associated with decrease of TET gene expression and 5-methylcytosine hydroxylation. Oncogene..

[23] Jin SG, Jiang Y, Qiu R, Rauch TA, Wang Y, Schackert G (2011). 5-Hydroxymethylcytosine is strongly depleted in human cancers but its levels do not correlate with IDH1 mutations. Cancer Res..

[24] Udali S, Guarini P, Moruzzi S, Ruzzenente A, Tammen SA, Guglielmi A et al. Global DNA methylation and hydroxymethylation differ in hepatocellular carcinoma and cholangiocarcinoma and relate to survival rate. Hepatology. 2015. doi:10.1002/hep.27823 10.1002/hep.27823 25833413

[25] Tang Y, Zheng SJ, Qi CB, Feng YQ, Yuan BF (2015). Sensitive and simultaneous determination of 5-methylcytosine and its oxidation products in genomic DNA by chemical derivatization coupled with liquid chromatography-tandem mass spectrometry analysis. Anal Chem..

[26] Yuan BF, Feng YQ (2014). Recent advances in the analysis of 5-methylcytosine and its oxidation products. TrAC-Trend Anal Chem..

[27] Song CX, Szulwach KE, Fu Y, Dai Q, Yi C, Li X (2011). Selective chemical labeling reveals the genome-wide distribution of 5-hydroxymethylcytosine. Nat Biotechnol..

[28] van den Hove DL, Chouliaras L, Rutten BP (2012). The role of 5-hydroxymethylcytosine in aging and Alzheimer's disease: current status and prospects for future studies. Curr Alzheimer Res..

[29] Ito S, Shen L, Dai Q, Wu SC, Collins LB, Swenberg JA (2011). Tet proteins can convert 5-methylcytosine to 5-formylcytosine and 5-carboxylcytosine. Science..

[30] Chen ML, Zhang J, Zhang Z, Yuan BF, Yu QW, Feng YQ (2013). Facile preparation of organic-silica hybrid monolith for capillary hydrophilic liquid chromatography based on "thiol-ene" click chemistry. J Chromatogr A..

[31] Jiang HP, Zhu JX, Peng C, Gao J, Zheng F, Xiao YX (2014). Facile one-pot synthesis of a aptamer-based organic-silica hybrid monolithic capillary column by "thiol-ene" click chemistry for detection of enantiomers of chemotherapeutic anthracyclines. Analyst..

[32] Jiang HP, Qi CB, Chu JM, Yuan BF, Feng YQ (2015). Profiling of cis-Diol-containing nucleosides and ribosylated metabolites by boronate-affinity organic-silica hybrid monolithic capillary liquid chromatography/mass spectrometry. Sci Rep..

[33] Song LG, James SR, Kazim L, Karpf AR (2005). Specific method for the determination of genomic DNA methylation by liquid chromatography-electrospray ionization tandem mass spectrometry. Anal Chem..

[34] Liu S, Wang J, Su YJ, Guerrero C, Zeng YX, Mitra D (2013). Quantitative assessment of Tet-induced oxidation products of 5-methylcytosine in cellular and tissue DNA. Nucleic Acids Res..

[35] Laird A, Thomson JP, Harrison DJ, Meehan RR (2013). 5-hydroxymethylcytosine profiling as an indicator of cellular state. Epigenomics-Uk..

[36] Wilson VL, Smith RA, Ma S, Cutler RG (1987). Genomic 5-methyldeoxycytidine decreases with age. J Biol Chem..

[37] Bocklandt S, Lin W, Sehl ME, Sanchez FJ, Sinsheimer JS, Horvath S (2011). Epigenetic predictor of age. PLoS One..

[38] Weidner CI, Lin Q, Koch CM, Eisele L, Beier F, Ziegler P (2014). Aging of blood can be tracked by DNA methylation changes at just three CpG sites. Genome Biol..

[39] Murgatroyd C, Patchev AV, Wu Y, Micale V, Bockmuhl Y, Fischer D (2009). Dynamic DNA methylation programs persistent adverse effects of early-life stress. Nat Neurosci..

[40] Inoue A, Zhang Y (2011). Replication-dependent loss of 5-hydroxymethylcytosine in mouse preimplantation embryos. Science..

[41] Yuan BF (2014). 5-methylcytosine and its derivatives. Adv Clin Chem..

[42] Fan Y, Feng YQ, Da SL, Shi ZG (2004). Poly (methacrylic acid-ethylene glycol dimethacrylate) monolithic capillary for in-tube solid phase microextraction coupled to high performance liquid chromatography and its application to determination of basic drugs in human serum. Anal Chim Acta..

