# Supplementary material for: DNA hydroxymethylation age of human blood determined by capillary hydrophilic-interaction liquid chromatography/mass spectrometry
Source: Clin Epigenetics. 2015 Jul 23;7(1):72. doi: 10.1186/s13148-015-0109-x (PMC4511989; doi:10.1186/s13148-015-0109-x)
Supplement: Additional file 1: — Supplementary Data Table S1. Comparison of the developed on-line trapping/cHILIC/ESI-MS method with other methods. Supplementary Data Table S2. The preparation of the quality control (QC) samples with the synthesized 5-mC- and 5-hmC-containing oligodeoxynucleotides. Supplementary Data Table S3. Accuracy of the method for the detection of 5-mdC and 5-hmdC. Supplementary Data Table S4. Intra- and inter-day imprecision for the quantification of 5-mdC and 5-hmdC by on-line trapping/cHILIC/ESI-MS method. Supplementary Data Table S5. The comparison of on-line trapping/cHILIC/ESI-MS method with normal LC/MS method for the quantification of 5-mC and 5-hmC in genomic DNA from 18 blood samples. Supplementary Data Table S6. Measured contents of 5-mdC and 5-hmdC in genomic DNA of blood from 238 healthy persons (blood samples were collected from Zhongnan Hospital of Wuhan University, Hubei, China). Supplementary Data Table S7. Measured contents of 5-mdC and 5-hmdC in genomic DNA of blood samples from 172 healthy persons (blood samples were collected from the First Affiliated Hospital of Zhengzhou University, Henan, China). Supplementary Data Table S8. The Spearman correlation of the contents of 5-mC and 5-hmC with respect to blood cell composition. Supplementary Data Table S9. The qualitative and quantitative ions for the detection of nucleosides. Supplementary Data Figure S1. Characterizations of hydrophilic organic-silica hybrid monolith. Supplementary Data Figure S2. Extracted-ion chromatograms of nucleosides by on-line trapping/cHILIC/ESI-MS analysis. Supplementary Data Figure S3. Optimizations of the on-line trapping/cHILIC/ESI-MS conditions. Supplementary Data Figure S4. The linear regression of 5-hmC content in genomic DNA of blood with age. Supplementary Data Figure S5. Correlation analysis of DNA methylation and hydroxymethylation with age using blood samples from Henan province, China. Supplementary Data Figure S6. Chronological age (x-axis) versus DNA hydroxymethylati [file 13148_2015_109_MOESM1_ESM.doc]

Supporting Information

for

**DNA Hydroxymethylation Age of Human Blood Determined by Capillary Hydrophilic-Interaction Liquid Chromatography / Mass Spectrometry**

Jun Xiong,^†,1^ Han-Peng Jiang,^†,1^ Chun-Yan Peng,^†,2^ Qian-Yun Deng,^2^ Meng-Dan Lan,^1^ Huan Zeng,^1^ Fang Zheng,^2^,*Yu-Qi Feng,^1^,* Bi-Feng Yuan ^1^,*

^1^ Key Laboratory of Analytical Chemistry for Biology and Medicine (Ministry of Education), Department of Chemistry, Wuhan University, Wuhan 430072, China.

^2^ Center for Gene Diagnosis, Zhongnan Hospital of Wuhan University, Wuhan 430071, China.

†These authors contributed equally to this work.

Running Title: DNA Hydroxymethylation Age of Human Blood

* To whom correspondence should be addressed. Tel. +86-27-68755595; fax. +86-27-68755595. E-mail address: bfyuan@whu.edu.cn; yqfeng@whu.edu.cn; zhengfang@whu.edu.cn

**Supplemental Data Table S1.** Comparison of the developed on-line trapping/*c*HILIC/ESI-MS method with other methods.

| **Detection Methods** | **Samples analyzed** | **Analytes** | **LOD (fmol)** | **Reference** | **Year** |
| --- | --- | --- | --- | --- | --- |
| Stable isotope dilution LC-MS/MS | Human urine samples | 5-mdC | 0.025 |  | 2015 |
|  |  | 5-hmdC | 0.25 |  |  |
| LC-MS/MS | DNA from HEK293A cells | 5-hmdC | 7.8 |  | 2014 |
| LC-ESI-QTOF-MS | DNA from hepatocellular carcinoma tissues | 5-mdC | 0.06 |  | 2013 |
|  |  | 5-hmdC | 0.19 |  |  |
| SPE-LC-MS/MS | DNA from three human cell lines and seven yeast strains | 5-hmdC | 1.5 |  | 2013 |
| LC-ESI-MS/MS | DNA from 293T cells, mouse embryonic stem cells and mouse tissues | 5-hmdC | 2.5 |  | 2011 |
| LC-MS/MS | DNA from human neuroblastoma SK-N-AS cells | 5-mdC | 0.5 |  | 2011 |
| LC-ESI-MS/MS | DNA from human colon adenocarcinoma cell line HCT116 and HCT116 derivative line DKO | 5-mdC | 0.2 |  | 2005 |
| On-line trapping/*c*HILIC/ESI-MS | DNA from human blood cells | 5-mdC | 0.04 | Current Study | 2015 |
|  |  | 5-hmdC | 0.19 |  |  |

**Reference:**

1. Yin RC, Mo JZ, Lu ML, Wang HL. Detection of Human Urinary 5-Hydroxymethylcytosine by Stable Isotope Dilution HPLC-MS/MS Analysis. Analy Chem. 2015;87:1846-52

2. Tsuji M, Matsunaga H, Jinno D, Tsukamoto H, Suzuki N, Tomioka Y. A validated quantitative liquid chromatography-tandem quadrupole mass spectrometry method for monitoring isotopologues to evaluate global modified cytosine ratios in genomic DNA. J Chromatogr B. 2014;953:38-47

3. Chen ML, Shen F, Huang W, Qi JH, Wang Y, Feng YQ et al. Quantification of 5-methylcytosine and 5-hydroxymethylcytosine in genomic DNA from hepatocellular carcinoma tissues by capillary hydrophilic-interaction liquid chromatography/quadrupole TOF mass spectrometry. Clin Chem. 2013;59:824-32

4. Tang Y, Chu JM, Huang W, Xiong J, Xing XW, Zhou X et al. Hydrophilic Material for the Selective Enrichment of 5-Hydroxymethylcytosine and Its Liquid Chromatography-Tandem Mass Spectrometry Detection. Anal Chem. 2013;85:6129-35

5. Ito S, Shen L, Dai Q, Wu SC, Collins LB, Swenberg JA et al. Tet proteins can convert 5-methylcytosine to 5-formylcytosine and 5-carboxylcytosine. Science. 2011;333:1300-3

6. Wang X, Suo Y, Yin R, Shen H, Wang H. Ultra-performance liquid chromatography/tandem mass spectrometry for accurate quantification of global DNA methylation in human sperms. J Chromatogr B. 2011;879:1647-52

7. Song LG, James SR, Kazim L, Karpf AR. Specific method for the determination of genomic DNA methylation by liquid chromatography-electrospray ionization tandem mass spectrometry. Anal Chem. 2005;77:504-10

**Supplemental Data Table S2.** The preparation of the quality control (QC) samples with the synthesized 5-mC- and 5-hmC-containing oligodeoxynucleotides.

| QC samples | | Molar ratio (%) | | |
| --- | --- | --- | --- | --- |
|  |  | DNA standard 1 | DNA Standard 2 | DNA standard 3 |
| 5-mC (%) | 1.00 (Low) | 94.9 | 5.1 |  |
|  | 3.00 (Medium) | 82.1 | 17.9 |  |
|  | 6.00 (High) | 51.3 | 48.7 |  |
| 5-hmC (%) | 0.0050 (Low) | 99.975 |  | 0.025 |
|  | 0.0100 (Medium) | 99.950 |  | 0.050 |
|  | 0.0500 (High) | 99.750 |  | 0.250 |
| DNA standard 1 (17-mer DNA) | | 5’-GGTACCCGCGGCTTAAG-3’ | | |
| DNA standard 2 (16-mer DNA) | | 5’-CCTCATCA(5-mC)GACCTAC-3’ | | |
| DNA standard 3 (17-mer DNA) | | 5’-CTTAAGCCG(5-hmC)GGGTACC-3’ | | |

**Supplemental Data Table S3.** Accuracy of the method for the detection of 5-mdC and 5-hmdC.

| QCs | Nominal | Observed | RSD % (n=3) | Relative error (%) |
| --- | --- | --- | --- | --- |
| 5-mdC (vs. [dC], %) | 1.00 | 1.12 | 6.5 | 12.0 |
|  | 3.00 | 3.17 | 7.4 | 5.7 |
|  | 6.00 | 6.26 | 9.6 | 4.3 |
| 5-hmdC (vs. [dC], %) | 0.0050 | 0.0057 | 10.1 | 14.0 |
|  | 0.0100 | 0.0109 | 2.7 | 9.0 |
|  | 0.0500 | 0.0454 | 11.2 | -9.2 |

**Supplemental Data Table S4.** Intra- and inter-day imprecision for the quantification of 5-mdC and 5-hmdC by on-line trapping/*c*HILIC/ESI-MS method.

|  | Intra-day (RSD %, n = 3) | | | Inter-day (RSD %, n = 5) | | |
| --- | --- | --- | --- | --- | --- | --- |
|  | 5-mdC/dC, 0.5% | 5-mdC/dC, 1.0% | 5-mdC/dC, 5.0% | 5-mdC/dC, 0.5% | 5-mdC/dC, 1.0% | 5-mdC/dC, 5.0% |
| 5-mdC | 3.1 | 1.9 | 2.6 | 3.4 | 7.8 | 7.3 |
|  | 5-hmdC/dC, 0.01% | 5-hmdC/dC, 0.05% | 5-hmdC/dC, 0.10% | 5-hmdC/dC, 0.01% | 5-hmdC/dC, 0.05% | 5-hmdC/dC, 0.10% |
| 5-hmdC | 10.4 | 8.4 | 9.1 | 13.2 | 9.1 | 11.4 |

**Supplemental Data Table S5.** The comparison of on-line trapping/*c*HILIC/ESI-MS method with normal LC/MS method for the quantification of 5-mC and 5-hmC in genomic DNA from 18 blood samples (the sample numbers are identical to that shown in Table S6).

| No. | Age | On-line trapping/*c*HILIC/ESI-MS | |  | Normal LC/MS | | | |
| --- | --- | --- | --- | --- | --- | --- | --- | --- |
|  |  | 5-mC | 5-hmC |  | 5-mC | Relative errors (%) | 5-hmC | Relative errors (%) |
| 10 | 4 | 3.14 ± 0.14 | 0.0309 ± 0.0007 |  | 3.58 ± 0.05 | 12.3 | 0.0282 ± 0.0000 | -9.6 |
| 11 | 5 | 3.38 ± 0.05 | 0.0284 ± 0.0015 |  | 4.01 ± 0.13 | 15.7 | 0.0301 ± 0.0000 | 5.6 |
| 15 | 6 | 3.27 ± 0.10 | 0.0326 ± 0.0010 |  | 3.74 ± 0.01 | 12.6 | 0.0290 ± 0.0025 | -12.4 |
| 49 | 20 | 3.61 ± 0.15 | 0.0247 ± 0.0008 |  | 3.76 ± 0.05 | 4.0 | 0.0232 ± 0.0004 | -6.5 |
| 54 | 21 | 3.13 ± 0.06 | 0.0203 ± 0.0006 |  | 3.31 ± 0.06 | 5.4 | 0.0196 ± 0.0009 | -3.4 |
| 57 | 22 | 3.37 ± 0.17 | 0.0269 ± 0.0008 |  | 3.15 ± 0.02 | -7.0 | 0.0259 ± 0.0008 | -3.9 |
| 60 | 23 | 2.99 ± 0.03 | 0.0195 ± 0.0002 |  | 2.95 ± 0.03 | -1.4 | 0.0228 ± 0.0018 | -2.0 |
| 62 | 24 | 3.71 ± 0.06 | 0.0262 ± 0.0008 |  | 3.67 ± 0.04 | -1.1 | 0.0242 ± 0.0026 | -8.3 |
| 137 | 49 | 3.04 ± 0.06 | 0.0138 ± 0.0009 |  | 3.19 ± 0.03 | 4.7 | 0.0132 ± 0.0022 | -4.6 |
| 143 | 51 | 3.33 ± 0.08 | 0.0136 ± 0.0002 |  | 3.44 ± 0.05 | 3.2 | 0.0142 ± 0.0008 | 4.2 |
| 150 | 53 | 3.65 ± 0.05 | 0.0122 ± 0.0005 |  | 3.40 ± 0.02 | -7.4 | 0.0124 ± 0.0015 | 1.6 |
| 154 | 54 | 3.27 ± 0.04 | 0.0111 ± 0.0006 |  | 3.43 ± 0.03 | 4.7 | 0.0121 ± 0.0000 | 8.3 |
| 156 | 55 | 3.18 ± 0.06 | 0.0134 ± 0.0008 |  | 3.21 ± 0.03 | 0.9 | 0.0136 ± 0.0011 | 1.5 |
| 193 | 65 | 3.50 ± 0.25 | 0.0121 ± 0.0001 |  | 3.78 ± 0.05 | 7.4 | 0.0133 ± 0.0004 | 9.0 |
| 194 | 65 | 3.16 ± 0.11 | 0.0102 ± 0.0000 |  | 3.20 ± 0.01 | 1.3 | 0.0112 ± 0.0009 | 8.9 |
| 200 | 67 | 3.64 ± 0.02 | 0.0115 ± 0.0002 |  | 3.47 ± 0.06 | -4.9 | 0.0129 ± 0.0001 | 10.9 |
| 208 | 70 | 3.14 ± 0.03 | 0.0113 ± 0.0006 |  | 3.34 ± 0.03 | 6.0 | 0.0104 ± 0.0011 | -8.7 |
| 214 | 72 | 3.23 ± 0.01 | 0.0085 ± 0.0004 |  | 3.33 ± 0.02 | 3.0 | 0.0082 ± 0.0005 | -3.7 |

**Supplemental Data Table S6.** Measured contents of 5-mdC and 5-hmdC in genomic DNA of blood from 238 healthy persons (blood samples were collected from Zhongnan Hospital of Wuhan University, Hubei, China).

| No. | Age (year**s**) | Gender | [5-mdC]/[dC+5-mdC+5-hmdC] (%) | [5-hmdC]/[ dC+5-mdC+5-hmdC] (%) |
| --- | --- | --- | --- | --- |
| 1 | 1 | Female | 3.26 ± 0.01 | 0.0287 ± 0.0016 |
| 2 | 2 | Female | 3.36 ± 0.02 | 0.0266 ± 0.0025 |
| 3 | 2 | Female | 3.22 ± 0.07 | 0.0261 ± 0.0025 |
| 4 | 2 | Male | 2.87 ± 0.14 | 0.0233 ± 0.0011 |
| 5 | 2 | Male | 3.67 ± 0.18 | 0.0211 ± 0.0006 |
| 6 | 2 | Female | 3.74 ± 0.00 | 0.0247 ± 0.0020 |
| 7 | 3 | Male | 3.08 ± 0.23 | 0.0254 ± 0.0033 |
| 8 | 3 | Male | 3.21 ± 0.21 | 0.0232 ± 0.0003 |
| 9 | 4 | Female | 3.85 ± 0.09 | 0.0255 ± 0.0040 |
| 10 | 4 | Female | 3.14 ± 0.14 | 0.0309 ± 0.0007 |
| 11 | 5 | Male | 3.38 ± 0.05 | 0.0284 ± 0.0015 |
| 12 | 5 | Male | 3.76 ± 0.12 | 0.0177 ± 0.0006 |
| 13 | 6 | Male | 3.20 ± 0.02 | 0.0289 ± 0.0003 |
| 14 | 6 | Female | 3.01 ± 0.09 | 0.0236± 0.0009 |
| 15 | 6 | Male | 3.27 ± 0.10 | 0.0326 ± 0.0010 |
| 16 | 7 | Male | 3.61 ± 0.04 | 0.0265 ± 0.0011 |
| 17 | 7 | Female | 3.42 ± 0.11 | 0.0269 ± 0.0005 |
| 18 | 8 | Female | 2.85 ± 0.08 | 0.0301 ± 0.0011 |
| 19 | 9 | Female | 3.26 ± 0.29 | 0.0260 ± 0.0018 |
| 20 | 10 | Female | 3.01 ± 0.08 | 0.0227 ± 0.0013 |
| 21 | 10 | Female | 3.30 ± 0.23 | 0.0242 ± 0.0001 |
| 22 | 10 | Female | 3.02 ± 0.10 | 0.0242 ± 0.0016 |
| 23 | 11 | Male | 3.23 ± 0.02 | 0.0228 ± 0.0001 |
| 24 | 11 | Female | 3.30 ± 0.08 | 0.0206 ± 0.0008 |
| 25 | 12 | Female | 3.42 ± 0.10 | 0.0266 ± 0.0007 |
| 26 | 12 | Male | 3.03 ± 0.02 | 0.0255 ± 0.0022 |
| 27 | 12 | Male | 3.16 ± 0.01 | 0.0240 ± 0.0004 |
| 28 | 13 | Male | 3.33 ± 0.08 | 0.0185 ± 0.0005 |
| 29 | 13 | Male | 2.88 ± 0.10 | 0.0261 ± 0.0006 |
| 30 | 14 | Female | 3.09 ± 0.18 | 0.0288 ± 0.0005 |
| 31 | 14 | Female | 3.75 ± 0.16 | 0.0218 ± 0.0004 |
| 32 | 14 | Male | 3.63 ± 0.07 | 0.0202 ± 0.0002 |
| 33 | 15 | Male | 3.21 ± 0.23 | 0.0273 ± 0.0013 |
| 34 | 15 | Male | 2.99 ± 0.15 | 0.0298 ± 0.0005 |
| 35 | 15 | Female | 3.58 ± 0.04 | 0.0181 ± 0.0007 |
| 36 | 16 | Female | 3.40 ± 0.07 | 0.0179 ± 0.0005 |
| 37 | 16 | Male | 3.22 ± 0.07 | 0.0253 ± 0.0013 |
| 38 | 17 | Female | 3.26 ± 0.01 | 0.0135 ± 0.0006 |
| 39 | 17 | Female | 3.08 ± 0.02 | 0.0273 ± 0.0006 |
| 40 | 17 | Female | 3.16 ± 0.10 | 0.0183 ± 0.0003 |
| 41 | 17 | Male | 3.12 ± 0.04 | 0.0242 ± 0.0024 |
| 42 | 18 | Female | 2.97 ± 0.11 | 0.0275 ± 0.0075 |
| 43 | 18 | Female | 3.13 ± 0.06 | 0.0203 ± 0.0027 |
| 44 | 18 | Male | 3.27 ± 0.06 | 0.0199 ± 0.0002 |
| 45 | 18 | Male | 3.06 ± 0.06 | 0.0222 ± 0.0011 |
| 46 | 19 | Female | 3.55 ± 0.27 | 0.0142 ± 0.0005 |
| 47 | 19 | Female | 3.19 ± 0.06 | 0.0297 ± 0.0010 |
| 48 | 19 | Female | 3.62 ± 0.01 | 0.0180 ± 0.0004 |
| 49 | 20 | Male | 3.61 ± 0.15 | 0.0246 ± 0.0008 |
| 50 | 20 | Female | 2.85 ± 0.11 | 0.0216 ± 0.0010 |
| 51 | 20 | Female | 3.47 ± 0.08 | 0.0165 ± 0.0003 |
| 52 | 20 | Male | 3.71 ± 0.05 | 0.0185 ± 0.0002 |
| 53 | 21 | Female | 3.68 ± 0.11 | 0.0256 ± 0.0004 |
| 54 | 21 | Male | 3.13 ± 0.06 | 0.0203 ± 0.0006 |
| 55 | 21 | Male | 3.08 ± 0.22 | 0.0271 ± 0.0007 |
| 56 | 22 | Female | 3.46 ± 0.24 | 0.0273 ± 0.0007 |
| 57 | 22 | Female | 3.37 ± 0.17 | 0.0268 ± 0.0008 |
| 58 | 22 | Male | 2.86 ± 0.06 | 0.0254 ± 0.0008 |
| 59 | 23 | Female | 3.34 ± 0.03 | 0.0161 ± 0.0005 |
| 60 | 23 | Male | 2.99 ± 0.03 | 0.0195 ± 0.0002 |
| 61 | 23 | Female | 3.79 ± 0.05 | 0.0195 ± 0.0003 |
| 62 | 24 | Female | 3.71 ± 0.06 | 0.0262 ± 0.0008 |
| 63 | 24 | Male | 3.26 ± 0.05 | 0.0221 ± 0.0012 |
| 64 | 24 | Female | 3.06 ± 0.06 | 0.0207 ± 0.0002 |
| 65 | 25 | Male | 3.80 ± 0.13 | 0.0218 ± 0.0006 |
| 66 | 25 | Female | 3.68 ± 0.09 | 0.0246 ± 0.0007 |
| 67 | 25 | Female | 3.76 ± 0.02 | 0.0155 ± 0.0011 |
| 68 | 26 | Male | 3.89 ± 0.06 | 0.0204 ± 0.0003 |
| 69 | 26 | Female | 3.78 ± 0.06 | 0.0201 ± 0.0005 |
| 70 | 26 | Male | 3.70 ± 0.15 | 0.0170 ± 0.0018 |
| 71 | 27 | Female | 3.50 ± 0.06 | 0.0199 ± 0.0012 |
| 72 | 27 | Male | 3.70 ± 0.03 | 0.0156 ± 0.0003 |
| 73 | 27 | Male | 3.13 ± 0.01 | 0.0195 ± 0.0001 |
| 74 | 28 | Male | 3.38 ± 0.03 | 0.0200 ± 0.0001 |
| 75 | 28 | Female | 2.82 ± 0.02 | 0.0201 ± 0.0006 |
| 76 | 28 | Female | 3.39 ± 0.03 | 0.0225 ± 0.0002 |
| 77 | 29 | Male | 3.68 ± 0.06 | 0.0176 ± 0.0008 |
| 78 | 29 | Female | 3.25 ± 0.12 | 0.0229 ± 0.0018 |
| 79 | 29 | Male | 3.10 ± 0.01 | 0.0269 ± 0.0007 |
| 80 | 30 | Male | 2.97 ± 0.05 | 0.0213 ± 0.0002 |
| 81 | 30 | Female | 2.99 ± 0.08 | 0.0206 ± 0.0002 |
| 82 | 30 | Female | 3.27 ± 0.07 | 0.0203 ± 0.0004 |
| 83 | 31 | Female | 3.29 ± 0.27 | 0.0189 ± 0.0004 |
| 84 | 31 | Male | 3.18 ± 0.26 | 0.0181 ± 0.0035 |
| 85 | 31 | Female | 2.98 ± 0.02 | 0.0189 ± 0.0003 |
| 86 | 32 | Male | 3.69 ± 0.12 | 0.0185 ± 0.0006 |
| 87 | 32 | Female | 3.15 ± 0.07 | 0.0178 ± 0.0002 |
| 88 | 32 | Female | 3.18 ± 0.13 | 0.0166 ± 0.0010 |
| 89 | 33 | Male | 3.18 ± 0.11 | 0.0160 ± 0.0001 |
| 90 | 33 | Male | 3.49 ± 0.06 | 0.0174 ± 0.0008 |
| 91 | 34 | Female | 3.11 ± 0.02 | 0.0232 ± 0.0012 |
| 92 | 34 | Male | 3.42 ± 0.14 | 0.0178 ± 0.0013 |
| 93 | 34 | Female | 3.61 ± 0.03 | 0.0155 ± 0.0008 |
| 94 | 35 | Male | 3.19 ± 0.05 | 0.0220 ± 0.0002 |
| 95 | 35 | Female | 3.43 ± 0.03 | 0.0157 ± 0.0006 |
| 96 | 35 | Female | 3.32 ± 0.18 | 0.0186 ± 0.0014 |
| 97 | 36 | Male | 3.05 ± 0.02 | 0.0182 ± 0.0010 |
| 98 | 36 | Female | 3.27 ± 0.01 | 0.0168 ± 0.0005 |
| 99 | 36 | Female | 3.38 ± 0.09 | 0.0162 ± 0.0001 |
| 100 | 37 | Female | 3.77 ± 0.05 | 0.0148 ± 0.0006 |
| 101 | 37 | Male | 3.16 ± 0.04 | 0.0192 ± 0.0008 |
| 102 | 37 | Female | 2.98 ± 0.11 | 0.0164 ± 0.0008 |
| 103 | 38 | Male | 3.17 ± 0.05 | 0.0166 ± 0.0005 |
| 104 | 38 | Female | 3.55 ± 0.00 | 0.0144 ± 0.0009 |
| 105 | 38 | Male | 3.20 ± 0.01 | 0.0172 ± 0.0006 |
| 106 | 39 | Male | 2.94 ± 0.09 | 0.0217 ± 0.0010 |
| 107 | 39 | Female | 3.21 ± 0.04 | 0.0166 ± 0.0004 |
| 108 | 39 | Male | 3.18 ± 0.10 | 0.0157 ± 0.0002 |
| 109 | 40 | Female | 3.54 ± 0.06 | 0.0181 ± 0.0002 |
| 110 | 40 | Male | 2.83 ± 0.03 | 0.0158 ± 0.0007 |
| 111 | 40 | Male | 3.09 ± 0.05 | 0.0138 ± 0.0003 |
| 112 | 41 | Male | 3.03 ± 0.04 | 0.0189 ± 0.0012 |
| 113 | 41 | Female | 2.87 ± 0.05 | 0.0117 ± 0.0009 |
| 114 | 41 | Female | 3.12 ± 0.09 | 0.0127 ± 0.0004 |
| 115 | 42 | Female | 3.45 ± 0.02 | 0.0183 ± 0.0002 |
| 116 | 42 | Male | 2.97 ± 0.10 | 0.0166 ± 0.0009 |
| 117 | 42 | Female | 2.79 ± 0.13 | 0.0220 ± 0.0003 |
| 118 | 43 | Female | 3.34 ± 0.05 | 0.0148 ± 0.0009 |
| 119 | 43 | Male | 3.41 ± 0.04 | 0.0172 ± 0.0009 |
| 120 | 44 | Male | 3.04 ± 0.04 | 0.0193 ± 0.0010 |
| 121 | 44 | Female | 3.58 ± 0.06 | 0.0163 ± 0.0006 |
| 122 | 44 | Female | 3.06 ± 0.06 | 0.0175 ± 0.0007 |
| 123 | 45 | Male | 3.79 ± 0.06 | 0.0128 ± 0.0003 |
| 124 | 45 | Female | 3.13 ± 0.06 | 0.0157 ± 0.0005 |
| 125 | 46 | Male | 3.19 ± 0.06 | 0.0113 ± 0.0008 |
| 126 | 46 | Female | 3.23 ± 0.04 | 0.0141 ± 0.0003 |
| 127 | 46 | Female | 3.56 ± 0.06 | 0.0114 ± 0.0003 |
| 128 | 47 | Male | 3.31 ± 0.05 | 0.0135 ± 0.0004 |
| 129 | 47 | Male | 2.99 ± 0.06 | 0.0116 ± 0.0003 |
| 130 | 47 | Female | 3.13 ± 0.04 | 0.0111 ± 0.0001 |
| 131 | 48 | Male | 2.95 ± 0.07 | 0.0112 ± 0.0003 |
| 132 | 48 | Female | 3.17 ± 0.02 | 0.0123 ± 0.0003 |
| 133 | 48 | Female | 3.00 ± 0.02 | 0.0120 ± 0.0003 |
| 134 | 48 | Female | 2.98 ± 0.12 | 0.0097 ± 0.0006 |
| 135 | 48 | Female | 3.68 ± 0.03 | 0.0108 ± 0.0008 |
| 136 | 49 | Male | 3.31 ± 0.10 | 0.0120 ± 0.0005 |
| 137 | 49 | Male | 3.04 ± 0.06 | 0.0138 ± 0.0009 |
| 138 | 50 | Male | 3.00 ± 0.03 | 0.0097 ± 0.0003 |
| 139 | 50 | Female | 2.98 ± 0.11 | 0.0153 ± 0.0004 |
| 140 | 50 | Male | 2.89 ± 0.06 | 0.0174 ± 0.0013 |
| 141 | 51 | Male | 3.01 ± 0.03 | 0.0130 ± 0.0004 |
| 142 | 51 | Male | 3.22 ± 0.04 | 0.0120 ± 0.0003 |
| 143 | 51 | Female | 3.33 ± 0.08 | 0.0135 ± 0.0002 |
| 144 | 52 | Male | 3.41 ± 0.04 | 0.0079 ± 0.0005 |
| 145 | 52 | Female | 3.80 ± 0.15 | 0.0102 ± 0.0002 |
| 146 | 52 | Female | 3.08 ± 0.02 | 0.0124 ± 0.0001 |
| 147 | 52 | Female | 3.15 ± 0.01 | 0.0168 ± 0.0005 |
| 148 | 52 | Female | 3.56 ± 0.07 | 0.0186 ± 0.0003 |
| 149 | 53 | Female | 2.92 ± 0.06 | 0.0116 ± 0.0006 |
| 150 | 53 | Male | 3.65 ± 0.05 | 0.0122 ± 0.0005 |
| 151 | 53 | Male | 3.40 ± 0.01 | 0.0146 ± 0.0007 |
| 152 | 54 | Female | 3.20 ± 0.03 | 0.0090 ± 0.0004 |
| 153 | 54 | Male | 3.15 ± 0.05 | 0.0112 ± 0.0005 |
| 154 | 54 | Female | 3.27 ± 0.04 | 0.0111 ± 0.0006 |
| 155 | 54 | Male | 3.56 ± 0.16 | 0.0149 ± 0.0014 |
| 156 | 55 | Female | 3.18 ± 0.011 | 0.0136 ± 0.0010 |
| 157 | 55 | Male | 3.26 ± 0.00 | 0.0126 ± 0.0003 |
| 158 | 55 | Female | 3.18 ± 0.00 | 0.0133 ± 0.0008 |
| 159 | 55 | Female | 3.45 ± 0.23 | 0.0108 ± 0.0013 |
| 160 | 56 | Male | 2.96 ± 0.05 | 0.0128 ± 0.0000 |
| 161 | 56 | Male | 3.18 ± 0.17 | 0.0142 ± 0.0009 |
| 162 | 56 | Male | 3.37 ± 0.20 | 0.0113 ± 0.0003 |
| 163 | 56 | Male | 3.29 ± 0.09 | 0.0117 ± 0.0006 |
| 164 | 56 | Female | 2.82 ± 0.13 | 0.0155 ± 0.0019 |
| 165 | 57 | Male | 3.27 ± 0.12 | 0.0126 ± 0.0006 |
| 166 | 57 | Male | 3.21 ± 0.02 | 0.0117 ± 0.0001 |
| 167 | 57 | Female | 3.33 ± 0.07 | 0.0087 ± 0.0003 |
| 168 | 57 | Male | 2.96 ± 0.04 | 0.0105 ± 0.0007 |
| 169 | 58 | Male | 2.98 ± 0.11 | 0.0101 ± 0.0000 |
| 170 | 58 | Male | 2.96 ± 0.06 | 0.0105 ± 0.0004 |
| 171 | 58 | Male | 3.08 ± 0.01 | 0.0128 ± 0.0001 |
| 172 | 58 | Female | 3.22 ± 0.02 | 0.0122 ± 0.0011 |
| 173 | 59 | Male | 3.53 ± 0.08 | 0.0121 ± 0.0007 |
| 174 | 59 | Male | 3.41 ± 0.25 | 0.0122 ± 0.0002 |
| 175 | 59 | Male | 3.33 ± 0.18 | 0.0103 ± 0.0002 |
| 176 | 60 | Male | 3.38 ± 0.22 | 0.0116 ± 0.0004 |
| 177 | 60 | Female | 3.54 ± 0.08 | 0.0123 ± 0.0006 |
| 178 | 60 | Female | 3.51 ± 0.06 | 0.0124 ± 0.0011 |
| 179 | 61 | Female | 2.99 ± 0.01 | 0.0111 ± 0.0002 |
| 180 | 61 | Male | 3.33 ± 0.04 | 0.0124 ± 0.0001 |
| 181 | 61 | Male | 3.67 ± 0.09 | 0.0112 ± 0.0001 |
| 182 | 62 | Female | 3.28 ± 0.01 | 0.0086 ± 0.0004 |
| 183 | 62 | Male | 3.06 ± 0.07 | 0.0157 ± 0.0010 |
| 184 | 62 | Female | 3.20 ± 0.03 | 0.0098 ± 0.0005 |
| 185 | 62 | Female | 3.32 ± 0.15 | 0.0119 ± 0.0007 |
| 186 | 63 | Female | 3.66 ± 0.12 | 0.0124 ± 0.0006 |
| 187 | 63 | Male | 2.96 ± 0.09 | 0.0119 ± 0.0001 |
| 188 | 63 | Female | 2.90 ± 0.16 | 0.0140 ± 0.0007 |
| 189 | 64 | Male | 3.25 ± 0.03 | 0.0098 ± 0.0005 |
| 190 | 64 | Female | 2.90 ± 0.08 | 0.0122 ± 0.0003 |
| 191 | 64 | Male | 3.01 ± 0.04 | 0.0139 ± 0.0004 |
| 192 | 64 | Male | 3.65 ± 0.04 | 0.0111 ± 0.0003 |
| 193 | 65 | Female | 3.50 ± 0.25 | 0.0121 ± 0.0001 |
| 194 | 65 | Male | 3.16 ± 0.11 | 0.0102 ± 0.0000 |
| 195 | 65 | Male | 3.15 ± 0.01 | 0.0139 ± 0.0004 |
| 196 | 65 | Male | 3.13 ± 0.08 | 0.0122 ± 0.0007 |
| 197 | 66 | Male | 2.95 ± 0.12 | 0.0177 ± 0.0020 |
| 198 | 66 | Male | 3.30 ± 0.04 | 0.0129 ± 0.0003 |
| 199 | 66 | Female | 3.20 ± 0.05 | 0.0125 ± 0.0006 |
| 200 | 67 | Female | 3.64 ± 0.02 | 0.0115 ± 0.0002 |
| 201 | 67 | Male | 3.12 ± 0.13 | 0.0098 ± 0.0011 |
| 202 | 68 | Male | 3.10 ± 0.29 | 0.0100 ± 0.0009 |
| 203 | 68 | Male | 3.43 ± 0.05 | 0.0102 ± 0.0014 |
| 204 | 69 | Male | 3.44 ± 0.07 | 0.0111 ± 0.0003 |
| 205 | 69 | Female | 3.38 ± 0.03 | 0.0146 ± 0.0007 |
| 206 | 69 | Female | 3.57 ± 0.12 | 0.0125 ± 0.0006 |
| 207 | 69 | Female | 3.11 ± 0.05 | 0.0111 ± 0.0003 |
| 208 | 70 | Female | 3.14 ± 0.03 | 0.0113 ± 0.0006 |
| 209 | 70 | Female | 2.82 ± 0.12 | 0.0119 ± 0.0001 |
| 210 | 70 | Male | 2.98 ± 0.14 | 0.0104 ± 0.0002 |
| 211 | 71 | Male | 2.72 ± 0.17 | 0.0135 ± 0.0003 |
| 212 | 71 | Male | 2.65 ± 0.10 | 0.0086 ± 0.0004 |
| 213 | 71 | Male | 3.69 ± 0.02 | 0.0110 ± 0.0001 |
| 214 | 72 | Male | 3.23 ± 0.01 | 0.0085 ± 0.0004 |
| 215 | 72 | Male | 2.69 ± 0.13 | 0.0108 ± 0.0006 |
| 216 | 72 | Male | 2.85 ± 0.08 | 0.0131 ± 0.0007 |
| 217 | 72 | Female | 3.08 ± 0.04 | 0.0114 ± 0.0003 |
| 218 | 73 | Male | 3.48 ± 0.05 | 0.0093 ± 0.0003 |
| 219 | 73 | Female | 3.02 ± 0.11 | 0.0129 ± 0.0013 |
| 220 | 73 | Female | 3.10 ± 0.06 | 0.0140 ± 0.0009 |
| 221 | 74 | Male | 2.84 ± 0.09 | 0.0129 ± 0.0010 |
| 222 | 74 | Male | 3.42 ± 0.05 | 0.0114 ± 0.0009 |
| 223 | 75 | Male | 2.87 ± 0.01 | 0.0114 ± 0.0006 |
| 224 | 75 | Male | 3.36 ± 0.09 | 0.0106 ± 0.0006 |
| 225 | 76 | Female | 2.97 ± 0.08 | 0.0119 ± 0.0003 |
| 226 | 76 | Male | 2.53 ± 0.06 | 0.0102 ± 0.0003 |
| 227 | 77 | Male | 2.62 ± 0.08 | 0.0097 ± 0.0008 |
| 228 | 77 | Male | 2.80 ± 0.11 | 0.0094 ± 0.0013 |
| 229 | 78 | Male | 3.22 ± 0.05 | 0.0113 ± 0.0006 |
| 230 | 78 | Male | 2.92 ± 0.05 | 0.0063 ± 0.0002 |
| 231 | 79 | Female | 2.92 ± 0.05 | 0.0112 ± 0.0007 |
| 232 | 79 | Female | 3.35 ± 0.05 | 0.0107 ± 0.0004 |
| 233 | 80 | Male | 3.19 ± 0.03 | 0.0116 ± 0.0003 |
| 234 | 80 | Female | 3.35 ± 0.03 | 0.0093 ± 0.0002 |
| 235 | 81 | Female | 3.19 ± 0.04 | 0.0067 ± 0.0001 |
| 236 | 81 | Male | 2.98 ± 0.05 | 0.0071 ± 0.0001 |
| 237 | 81 | Female | 3.00 ± 0.09 | 0.0113 ± 0.0006 |
| 238 | 82 | Male | 3.46 ± 0.16 | 0.0102 ± 0.0008 |

**Supplemental Data Table S7.** Measured contents of 5-mdC and 5-hmdC in genomic DNA of blood samples from 172 healthy persons (blood samples were collected from the First Affiliated Hospital of Zhengzhou University, Henan, China). And cell compositions were measured. RBC, red blood cells; WBC, white blood cells; NEUT, neutrophil granulocytes; EO, eosinophile granulocytes; BASO, basophile granulocytes; LYMPH, lymphocytes; MONO, monocytes.

| No. | Age | Gender | 5-mdC/(dC + 5-mdC + 5-hmdC) (%) | 5-hmdC/(dC + 5-mdC + 5-hmdC) (%) | RBC | WBC | NEUT | EO | BASO | LYMPH | MONO |
| --- | --- | --- | --- | --- | --- | --- | --- | --- | --- | --- | --- |
| 1 | 3 | Female | 3.82 ± 0.05 | 0.0274 ± 0.0038 | 4.30 | 9.90 | 6.75 | 0.43 | 0.01 | 2.39 | 0.32 |
| 2 | 3 | Male | 3.82 ± 0.05 | 0.0335 ± 0.0010 | 4.34 | 7.54 | 3.88 | 0.39 | 0.04 | 2.84 | 0.39 |
| 3 | 4 | Male | 3.61 ± 0.27 | 0.0318 ± 0.0017 | 4.37 | 7.25 | 4.49 | 0.10 | 0.01 | 2.36 | 0.30 |
| 4 | 4 | Female | 3.68 ± 0.06 | 0.0276 ± 0.0029 | 4.09 | 8.74 | 5.70 | 0 | 0.02 | 2.83 | 0.19 |
| 5 | 5 | Male | 3.83 ± 0.14 | 0.0362 ± 0.0020 | 4.58 | 9.11 | 5.58 | 0.2 | 0.02 | 2.69 | 0.62 |
| 6 | 5 | Female | 4.12 ± 0.02 | 0.0356 ± 0.0037 | 5.23 | 7.11 | 4.52 | 0.07 | 0.05 | 2.07 | 0.40 |
| 7 | 6 | Male | 3.60 ± 0.00 | 0.0277 ± 0.0025 | 4.43 | 6.77 | 4.17 | 0.10 | 0.02 | 2.13 | 0.35 |
| 8 | 6 | Male | 3.05 ± 0.08 | 0.0225 ± 0.0012 | 4.55 | 8.45 | 4.51 | 0.16 | 0.04 | 3.29 | 0.45 |
| 9 | 7 | Female | 3.41 ± 0.12 | 0.0293 ± 0.0040 | 4.33 | 9.2 | 5.74 | 0.07 | 0.02 | 2.89 | 0.48 |
| 10 | 7 | Female | 3.51 ± 0.04 | 0.0283 ± 0.0024 | 3.88 | 4.48 | 2.57 | 0.17 | 0.02 | 1.53 | 0.19 |
| 11 | 8 | Female | 3.60 ± 0.20 | 0.0279 ± 0.0005 | 4.65 | 5.01 | 2.96 | 0.10 | 0.04 | 1.63 | 0.28 |
| 12 | 8 | Male | 3.40 ± 0.05 | 0.0231 ± 0.0005 | 4.90 | 9.08 | 4.99 | 0.08 | 0.08 | 3.52 | 0.40 |
| 13 | 9 | Female | 3.72 ± 0.09 | 0.0279 ± 0.0018 | 4.45 | 6.53 | 3.85 | 0.11 | 0.01 | 2.27 | 0.29 |
| 14 | 9 | Female | 3.04 ± 0.08 | 0.0250 ± 0.0029 | 4.22 | 5.77 | 3.62 | 0.04 | 0.01 | 1.81 | 0.29 |
| 15 | 10 | Male | 2.94 ± 0.01 | 0.0262 ± 0.0008 | 4.98 | 7.88 | 4.93 | 0.15 | 0.04 | 2.48 | 0.28 |
| 16 | 10 | Male | 3.54 ± 0.09 | 0.0282 ± 0.0010 | 4.14 | 7.93 | 5.17 | 0.13 | 0.03 | 2.17 | 0.43 |
| 17 | 11 | Male | 3.65 ± 0.24 | 0.0340 ± 0.0029 | 4.49 | 7.81 | 4.51 | 0.58 | 0.02 | 2.25 | 0.45 |
| 18 | 11 | Female | 3.40 ± 0.09 | 0.0274 ± 0.0013 | 4.23 | 4.65 | 2.84 | 0.09 | 0.05 | 1.43 | 0.24 |
| 19 | 12 | Female | 3.80 ± 0.04 | 0.0315 ± 0.0041 | 4.69 | 9.36 | 5.95 | 0.27 | 0.07 | 2.81 | 0.31 |
| 20 | 12 | Male | 2.88 ± 0.02 | 0.0207 ± 0.0020 | 4.11 | 4.47 | 2.34 | 0.10 | 0.01 | 1.75 | 0.27 |
| 21 | 13 | Male | 4.01 ± 0.26 | 0.0319 ± 0.0014 | 4.07 | 9.78 | 6.02 | 0.25 | 0.02 | 3.89 | 0.57 |
| 22 | 13 | Female | 3.39 ± 0.03 | 0.0295 ± 0.0001 | 4.53 | 4.72 | 2.54 | 0.13 | 0.02 | 1.64 | 0.39 |
| 23 | 14 | Male | 3.08 ± 0.03 | 0.0240 ± 0.0008 | 4.60 | 5.25 | 3.52 | 0.25 | 0.02 | 2.09 | 0.42 |
| 24 | 14 | Female | 3.08 ± 0.04 | 0.0267 ± 0.0019 | 4.28 | 8.77 | 5.26 | 0.31 | 0.03 | 2.63 | 0.54 |
| 25 | 18 | Female | 3.07 ± 0.09 | 0.0280 ± 0.0023 | 4.21 | 8.10 | 5.80 | 0.13 | 0.03 | 1.70 | 0.40 |
| 26 | 18 | Female | 3.31 ± 0.06 | 0.0261 ± 0.0006 | 4.55 | 5.07 | 2.40 | 0.09 | 0 | 2.00 | 0.60 |
| 27 | 19 | Female | 3.23 ± 0.16 | 0.0251 ± 0.0001 | 4.19 | 5.50 | 3.60 | 0.04 | 0.04 | 1.40 | 0.40 |
| 28 | 19 | Female | 3.11 ± 0.25 | 0.0264 ± 0.0036 | 4.45 | 7.40 | 4.80 | 0.05 | 0.04 | 2.00 | 0.50 |
| 29 | 20 | Female | 3.17 ± 0.04 | 0.0223 ± 0.0012 | 3.87 | 6.26 | 4.00 | 0.13 | 0.02 | 1.80 | 0.30 |
| 30 | 20 | Female | 3.45 ± 0.26 | 0.0215 ± 0.0002 | 4.51 | 5.69 | 2.50 | 0.45 | 0.03 | 2.20 | 0.50 |
| 31 | 21 | Female | 3.98 ± 0.11 | 0.0218 ± 0.0008 | 4.32 | 6.51 | 3.70 | 0.05 | 0.05 | 2.20 | 0.50 |
| 32 | 21 | Male | 3.24 ± 0.10 | 0.0249 ± 0.0007 | 4.90 | 8.08 | 5.00 | 0.18 | 0.04 | 2.40 | 0.50 |
| 33 | 21 | Female | 3.56 ± 0.01 | 0.0225 ± 0.0015 | 4.38 | 8.23 | 4.83 | 0.17 | 0.06 | 2.71 | 0.47 |
| 34 | 22 | Female | 3.49 ± 0.05 | 0.0200 ± 0.0000 | 4.21 | 5.60 | 2.80 | 0.03 | 0.02 | 2.40 | 0.40 |
| 35 | 22 | Male | 3.63 ± 0.16 | 0.0224 ± 0.0009 | 5.31 | 8.34 | 5.10 | 0.08 | 0.03 | 2.40 | 0.70 |
| 36 | 22 | Female | 3.41 ± 0.00 | 0.0223 ± 0.0012 | 4.28 | 5.80 | 3.51 | 0.15 | 0.05 | 1.85 | 0.24 |
| 37 | 23 | Female | 3.68 ± 0.21 | 0.0242 ± 0.0010 | 3.98 | 5.52 | 2.50 | 0.05 | 0.04 | 2.48 | 0.45 |
| 38 | 23 | Male | 3.82 ± 0.02 | 0.0245 ± 0.0016 | 4.81 | 5.40 | 3.20 | 0.16 | 0.03 | 1.60 | 0.40 |
| 39 | 23 | Female | 3.37 ± 0.15 | 0.0192 ± 0.0011 | 4.11 | 6.90 | 3.00 | 0.22 | 0.03 | 3.30 | 0.40 |
| 40 | 24 | Male | 3.39 ± 0.16 | 0.0218 ± 0.0010 | 5.25 | 6.30 | 3.00 | 0.13 | 0.01 | 2.50 | 0.70 |
| 41 | 24 | Female | 3.65 ± 0.02 | 0.0200 ± 0.0003 | 4.13 | 5.31 | 3.00 | 0.03 | 0.03 | 2.00 | 0.30 |
| 42 | 24 | Female | 3.50 ± 0.02 | 0.0215 ± 0.0007 | 4.56 | 7.16 | 3.56 | 0.47 | 0.02 | 2.59 | 0.53 |
| 43 | 25 | Female | 3.25 ± 0.01 | 0.0210 ± 0.0005 | 4.09 | 8.60 | 6.10 | 0.09 | 0.03 | 1.70 | 0.60 |
| 44 | 25 | Male | 3.38 ± 0.00 | 0.0200 ± 0.0006 | 5.21 | 6.40 | 4.10 | 0.03 | 0.03 | 1.70 | 0.50 |
| 45 | 26 | Male | 4.03 ± 0.25 | 0.0211 ± 0.0011 | 5.14 | 6.90 | 4.30 | 0.08 | 0.01 | 2.00 | 0.60 |
| 46 | 26 | Female | 3.52 ± 0.18 | 0.0249 ± 0.0007 | 4.13 | 6.00 | 3.70 | 0.01 | 0.03 | 2.00 | 0.30 |
| 47 | 27 | Female | 3.75 ± 0.07 | 0.0232 ± 0.0008 | 4.67 | 6.00 | 3.10 | 0.13 | 0.02 | 2.40 | 0.30 |
| 48 | 27 | Female | 3.23 ± 0.00 | 0.0188 ± 0.0001 | 4.40 | 5.40 | 2.90 | 0.04 | 0.02 | 2.10 | 0.20 |
| 49 | 27 | Male | 3.50 ± 0.08 | 0.0210 ± 0.0011 | 5.14 | 5.55 | 3.71 | 0.03 | 0.01 | 1.58 | 0.23 |
| 50 | 27 | Female | 3.38 ± 0.11 | 0.0205 ± 0.0006 | 3.80 | 5.14 | 3.16 | 0.07 | 0.01 | 1.64 | 0.27 |
| 51 | 28 | Female | 3.57 ± 0.07 | 0.0248 ± 0.0006 | 4.50 | 7.84 | 4.52 | 0.07 | 0.07 | 2.80 | 0.38 |
| 52 | 28 | Male | 3.18 ± 0.21 | 0.0221 ± 0.0031 | 4.37 | 4.70 | 2.40 | 0.07 | 0.03 | 1.90 | 0.30 |
| 53 | 28 | Female | 3.71 ± 0.02 | 0.0200 ± 0.0012 | 4.72 | 7.40 | 3.90 | 0.30 | 0.05 | 2.70 | 0.40 |
| 54 | 29 | Female | 3.49 ± 0.06 | 0.0216 ± 0.0003 | 3.99 | 5.63 | 3.50 | 0.05 | 0.02 | 1.70 | 0.40 |
| 55 | 29 | Female | 3.62 ± 0.17 | 0.0217 ± 0.0008 | 4.23 | 5.55 | 3.20 | 0.04 | 0.02 | 2.00 | 0.30 |
| 56 | 30 | Female | 3.58 ± 0.06 | 0.0185 ± 0.0001 | 4.64 | 6.00 | 3.40 | 0.08 | 0.03 | 2.10 | 0.40 |
| 57 | 30 | Female | 3.22 ± 0.05 | 0.0206 ± 0.0025 | 4.50 | 4.79 | 2.40 | 0.03 | 0 | 2.10 | 0.30 |
| 58 | 30 | Female | 3.51 ± 0.01 | 0.0222 ± 0.0013 | 4.12 | 6.43 | 2.59 | 0.18 | 0.04 | 3.19 | 0.44 |
| 59 | 30 | Male | 3.38 ± 0.03 | 0.0235 ± 0.0003 | 5.50 | 7.36 | 3.83 | 0.36 | 0.06 | 2.52 | 0.60 |
| 60 | 31 | Male | 3.68 ± 0.06 | 0.0175 ± 0.0016 | 4.67 | 6.99 | 3.70 | 0.39 | 0.03 | 2.40 | 0.50 |
| 61 | 31 | Male | 3.35 ± 0.14 | 0.0180 ± 0.0006 | 5.33 | 6.00 | 3.90 | 0.08 | 0.02 | 1.60 | 0.50 |
| 62 | 32 | Female | 3.10 ± 0.01 | 0.0186 ± 0.0006 | 4.51 | 4.60 | 2.50 | 0.11 | 0.02 | 1.60 | 0.40 |
| 63 | 32 | Female | 4.32 ± 0.01 | 0.0164 ± 0.0004 | 4.13 | 7.20 | 3.80 | 0.29 | 0.03 | 2.50 | 0.50 |
| 64 | 32 | Male | 3.68 ± 0.17 | 0.0197 ± 0.0005 | 5.34 | 6.39 | 3.70 | 0.11 | 0.04 | 2.05 | 0.49 |
| 65 | 32 | Female | 3.42 ± 0.12 | 0.0181 ± 0.0018 | 4.04 | 6.04 | 3.10 | 0.13 | 0.02 | 2.47 | 0.31 |
| 66 | 33 | Female | 3.41 ± 0.11 | 0.0212 ± 0.0031 | 4.27 | 5.03 | 2.79 | 0.04 | 0.02 | 1.82 | 0.37 |
| 67 | 33 | Female | 3.32 ± 0.11 | 0.0159 ± 0.0001 | 4.23 | 6.66 | 4.40 | 0.01 | 0.04 | 1.80 | 0.50 |
| 68 | 33 | Male | 3.45 ± 0.10 | 0.0197 ± 0.0012 | 5.26 | 6.68 | 4.30 | 0.09 | 0.04 | 1.80 | 0.50 |
| 69 | 34 | Male | 3.18 ± 0.23 | 0.0211 ± 0.0025 | 5.08 | 5.20 | 2.80 | 0.08 | 0.02 | 1.90 | 0.40 |
| 70 | 34 | Male | 3.31 ± 0.08 | 0.0200 ± 0.0018 | 5.28 | 7.00 | 4.30 | 0.04 | 0.02 | 2.10 | 0.40 |
| 71 | 34 | Female | 3.56 ± 0.23 | 0.0184 ± 0.0024 | 4.58 | 5.34 | 3.09 | 0.07 | 0.03 | 1.65 | 0.50 |
| 72 | 34 | Male | 3.28 ± 0.17 | 0.0167 ± 0.0013 | 4.70 | 4.61 | 2.73 | 0.13 | 0.03 | 1.46 | 0.26 |
| 73 | 35 | Male | 3.40 ± 0.08 | 0.0170 ± 0.0007 | 5.15 | 6.75 | 4.06 | 0.22 | 0.09 | 1.78 | 0.59 |
| 74 | 35 | Male | 2.84 ± 0.09 | 0.0179 ± 0.0008 | 5.31 | 7.90 | 4.90 | 0.16 | 0.02 | 2.30 | 0.50 |
| 75 | 35 | Female | 3.13 ± 0.06 | 0.0166 ± 0.0006 | 4.41 | 7.50 | 4.20 | 0.29 | 0.03 | 2.30 | 0.70 |
| 76 | 36 | Female | 3.02 ± 0.17 | 0.0170 ± 0.0004 | 4.73 | 7.10 | 4.10 | 0.42 | 0.03 | 2.20 | 0.40 |
| 77 | 36 | Female | 2.96 ± 0.05 | 0.0158 ± 0.0015 | 4.17 | 4.69 | 2.60 | 0.15 | 0.02 | 1.50 | 0.50 |
| 78 | 37 | Male | 3.30 ± 0.06 | 0.0202 ± 0.0019 | 5.52 | 6.70 | 3.70 | 0.12 | 0.03 | 2.50 | 0.40 |
| 79 | 37 | Female | 3.63 ± 0.10 | 0.0183 ± 0.0013 | 4.28 | 6.75 | 3.77 | 0.04 | 0.04 | 2.41 | 0.48 |
| 80 | 37 | Male | 3.56 ± 0.16 | 0.0201 ± 0.0013 | 5.32 | 7.20 | 3.87 | 0.08 | 0.07 | 2.65 | 0.53 |
| 81 | 38 | Male | 3.59 ± 0.06 | 0.0179 ± 0.0010 | 4.88 | 8.76 | 5.80 | 0.13 | 0.06 | 2.39 | 0.38 |
| 82 | 38 | Male | 3.41 ± 0.01 | 0.0171 ± 0.0021 | 5.48 | 7.40 | 3.30 | 0.83 | 0.05 | 2.60 | 0.60 |
| 83 | 39 | Male | 3.32 ± 0.15 | 0.0180 ± 0.0031 | 5.17 | 7.80 | 3.70 | 0.07 | 0.03 | 3.50 | 0.40 |
| 84 | 39 | Female | 3.26 ± 0.02 | 0.0164 ± 0.0001 | 4.07 | 8.00 | 5.00 | 0.12 | 0.06 | 2.40 | 0.50 |
| 85 | 39 | Female | 3.42 ± 0.02 | 0.0172 ± 0.0031 | 4.44 | 5.52 | 2.64 | 0.06 | 0.04 | 2.15 | 0.63 |
| 86 | 40 | Male | 3.79 ± 0.07 | 0.0166 ± 0.0000 | 5.50 | 8.98 | 4.66 | 0.30 | 0.10 | 3.36 | 0.55 |
| 87 | 40 | Female | 3.26 ± 0.01 | 0.0154 ± 0.0008 | 4.65 | 7.10 | 4.00 | 0.04 | 0.03 | 2.70 | 0.40 |
| 88 | 40 | Female | 3.82 ± 0.07 | 0.0213 ± 0.0003 | 4.13 | 4.40 | 2.90 | 0.02 | 0.03 | 1.00 | 0.40 |
| 89 | 41 | Male | 3.00 ± 0.12 | 0.0219 ± 0.0000 | 5.49 | 7.10 | 4.30 | 0.06 | 0.05 | 2.20 | 0.50 |
| 90 | 41 | Female | 4.22 ± 0.17 | 0.0165 ± 0.0003 | 4.49 | 7.00 | 4.80 | 0.20 | 0.06 | 1.60 | 0.30 |
| 91 | 41 | Female | 3.35 ± 0.01 | 0.0152 ± 0.0022 | 4.43 | 6.83 | 3.38 | 0.06 | 0.04 | 2.78 | 0.58 |
| 92 | 41 | Male | 3.48 ± 0.11 | 0.0203 ± 0.0006 | 5.58 | 5.71 | 2.72 | 0.10 | 0.04 | 2.33 | 0.52 |
| 93 | 42 | Female | 3.48 ± 0.03 | 0.0211 ± 0.0014 | 4.38 | 5.38 | 2.88 | 0.06 | 0.06 | 2.05 | 0.33 |
| 94 | 42 | Male | 3.30 ± 0.09 | 0.0165 ± 0.0019 | 5.93 | 5.52 | 3.00 | 0.11 | 0.04 | 2.16 | 0.21 |
| 95 | 42 | Female | 3.02 ± 0.07 | 0.0143 ± 0.0003 | 4.71 | 5.50 | 3.80 | 0.01 | 0.02 | 1.40 | 0.30 |
| 96 | 42 | Female | 3.45 ± 0.09 | 0.0195 ± 0.0012 | 4.10 | 7.62 | 4.80 | 0.18 | 0.05 | 2.10 | 0.50 |
| 97 | 43 | Male | 2.82 ± 0.18 | 0.0193 ± 0.0001 | 5.29 | 5.50 | 3.70 | 0.15 | 0.05 | 1.20 | 0.30 |
| 98 | 43 | Male | 3.20 ± 0.10 | 0.0213 ± 0.0024 | 5.02 | 9.26 | 6.10 | 0.47 | 0.06 | 2.00 | 0.70 |
| 99 | 43 | Male | 3.56 ± 0.15 | 0.0192 ± 0.0008 | 5.00 | 5.64 | 3.02 | 0.24 | 0.06 | 1.88 | 0.44 |
| 100 | 44 | Female | 3.38 ± 0.12 | 0.0166 ± 0.0013 | 4.26 | 4.76 | 2.39 | 0.14 | 0.03 | 1.92 | 0.27 |
| 101 | 44 | Male | 3.49 ± 0.11 | 0.0187 ± 0.0002 | 5.21 | 6.52 | 3.65 | 0.41 | 0.08 | 1.91 | 0.47 |
| 102 | 44 | Female | 3.59 ± 0.12 | 0.0184 ± 0.0021 | 4.17 | 6.50 | 3.80 | 0.04 | 0.02 | 2.10 | 0.50 |
| 103 | 44 | Female | 3.18 ± 0.22 | 0.0203 ± 0.0006 | 4.22 | 6.82 | 4.80 | 0.05 | 0.02 | 1.50 | 0.50 |
| 104 | 45 | Female | 3.25 ± 0.06 | 0.0168 ± 0.0008 | 4.20 | 8.20 | 5.60 | 0.03 | 0.03 | 2.10 | 0.50 |
| 105 | 45 | Male | 3.26 ± 0.07 | 0.0141 ± 0.0001 | 5.14 | 6.30 | 3.90 | 0.08 | 0.03 | 1.90 | 0.40 |
| 106 | 45 | Male | 3.37 ± 0.08 | 0.0161 ± 0.0007 | 5.07 | 4.91 | 2.41 | 0.11 | 0.04 | 1.97 | 0.37 |
| 107 | 46 | Male | 3.49 ± 0.09 | 0.0166 ± 0.0011 | 4.80 | 5.41 | 2.68 | 0.07 | 0.04 | 2.12 | 0.51 |
| 108 | 46 | Female | 3.58 ± 0.11 | 0.0151 ± 0.0026 | 4.55 | 5.31 | 2.43 | 0.23 | 0.06 | 2.14 | 0.44 |
| 109 | 47 | Female | 3.84 ± 0.03 | 0.0198 ± 0.0001 | 4.13 | 7.87 | 4.56 | 0.08 | 0.09 | 2.60 | 0.53 |
| 110 | 48 | Female | 3.58 ± 0.21 | 0.0160 ± 0.0013 | 4.26 | 5.99 | 3.98 | 0.16 | 0.04 | 1.42 | 0.39 |
| 111 | 48 | Male | 3.35 ± 0.05 | 0.0145 ± 0.0006 | 4.93 | 6.50 | 3.64 | 0.13 | 0.04 | 2.21 | 0.47 |
| 112 | 49 | Male | 3.65 ± 0.07 | 0.0182 ± 0.0002 | 6.01 | 6.45 | 2.69 | 0.16 | 0.07 | 3.08 | 0.45 |
| 113 | 49 | Female | 3.40 ± 0.24 | 0.0167 ± 0.0035 | 5.01 | 5.52 | 3.73 | 0.03 | 0 | 1.42 | 0.33 |
| 114 | 50 | Female | 3.02 ± 0.33 | 0.0181 ± 0.0015 | 4.49 | 7.14 | 3.41 | 0.24 | 0.09 | 2.79 | 0.61 |
| 115 | 50 | Male | 3.42 ± 0.11 | 0.0185 ± 0.0009 | 5.02 | 6.75 | 4.05 | 0.39 | 0.07 | 1.67 | 0.57 |
| 116 | 51 | Male | 3.09 ± 0.10 | 0.0138 ± 0.0013 | 4.26 | 6.45 | 3.40 | 0.27 | 0.06 | 2.26 | 0.46 |
| 117 | 51 | Female | 2.61 ± 0.07 | 0.0124 ± 0.0011 | 4.58 | 4.46 | 2.74 | 0.02 | 0.02 | 1.39 | 0.29 |
| 118 | 52 | Male | 3.69 ± 0.16 | 0.0198 ± 0.0025 | 4.55 | 6.39 | 4.03 | 0.10 | 0.06 | 1.75 | 0.45 |
| 119 | 52 | Female | 3.64 ± 0.29 | 0.0176 ± 0.0009 | 3.99 | 4.58 | 1.79 | 0.05 | 0.06 | 2.22 | 0.47 |
| 120 | 54 | Male | 3.69 ± 0.16 | 0.0187 ± 0.0001 | 4.38 | 5.45 | 3.09 | 0.11 | 0.03 | 1.87 | 0.36 |
| 121 | 55 | Female | 3.06 ± 0.05 | 0.0131 ± 0.0021 | 4.24 | 4.46 | 1.93 | 0.07 | 0.02 | 2.13 | 0.31 |
| 122 | 57 | Female | 3.94 ± 0.12 | 0.0188 ± 0.0021 | 4.49 | 5.60 | 2.60 | 0.23 | 0.05 | 2.33 | 0.39 |
| 123 | 58 | Male | 3.58 ± 0.11 | 0.0193 ± 0.0017 | 4.61 | 4.76 | 2.62 | 0.23 | 0.05 | 1.52 | 0.34 |
| 124 | 58 | Female | 3.21 ± 0.26 | 0.0167 ± 0.0042 | 4.45 | 5.50 | 2.92 | 0.14 | 0.03 | 1.91 | 0.50 |
| 125 | 59 | Male | 3.56 ± 0.21 | 0.0203 ± 0.0062 | 4.43 | 7.66 | 4.54 | 0.16 | 0.05 | 2.33 | 0.58 |
| 126 | 59 | Female | 3.34 ± 0.01 | 0.0124 ± 0.0013 | 4.29 | 6.62 | 3.87 | 0.33 | 0.04 | 1.8 | 0.58 |
| 127 | 60 | Female | 3.45 ± 0.07 | 0.0217 ± 0.0001 | 4.52 | 5.60 | 3.38 | 0.10 | 0.04 | 1.71 | 0.37 |
| 128 | 61 | Male | 3.66 ± 0.31 | 0.0169 ± 0.0003 | 5.09 | 8.71 | 4.84 | 0.32 | 0.07 | 2.94 | 0.54 |
| 129 | 62 | Male | 3.26 ± 0.04 | 0.0141 ± 0.0009 | 5.03 | 5.20 | 2.75 | 0.15 | 0.02 | 1.87 | 0.40 |
| 130 | 63 | Female | 3.34 ± 0.01 | 0.0138 ± 0.0005 | 4.22 | 5.02 | 2.52 | 0.03 | 0.04 | 2.15 | 0.26 |
| 131 | 63 | Male | 2.93 ± 0.09 | 0.0103 ± 0.0011 | 4.77 | 6.06 | 3.35 | 0.10 | 0.03 | 2.16 | 0.42 |
| 132 | 64 | Female | 3.54 ± 0.00 | 0.0157 ± 0.0030 | 4.51 | 5.90 | 3.61 | 0.11 | 0.05 | 1.70 | 0.44 |
| 133 | 64 | Female | 3.26 ± 0.02 | 0.0165 ± 0.0028 | 4.02 | 4.55 | 2.03 | 0.15 | 0.06 | 1.95 | 0.36 |
| 134 | 65 | Male | 2.92 ± 0.07 | 0.0115 ± 0.0001 | 4.22 | 4.72 | 2.08 | 0.05 | 0.01 | 2.28 | 0.29 |
| 135 | 65 | Male | 3.33 ± 0.09 | 0.0160 ± 0.0016 | 5.38 | 8.88 | 5.62 | 0.25 | 0 | 2.44 | 0.56 |
| 136 | 66 | Female | 3.10 ± 0.09 | 0.0137 ± 0.0008 | 4.00 | 3.70 | 2.31 | 0.04 | 0.04 | 1.14 | 0.18 |
| 137 | 66 | Female | 3.25 ± 0.03 | 0.0146 ± 0.0005 | 4.80 | 5.62 | 3.01 | 0.10 | 0.03 | 2.13 | 0.35 |
| 138 | 66 | Male | 3.20 ± 0.06 | 0.0162 ± 0.0021 | 5.32 | 6.22 | 3.11 | 0.37 | 0.07 | 2.22 | 0.45 |
| 139 | 66 | Female | 3.18 ± 0.27 | 0.0173 ± 0.0025 | 4.72 | 7.42 | 4.62 | 0.10 | 0.24 | 1.95 | 0.52 |
| 140 | 66 | Male | 2.95 ± 0.02 | 0.0143 ± 0.0024 | 4.48 | 5.50 | 3.49 | 0.27 | 0.05 | 1.22 | 0.47 |
| 141 | 67 | Male | 3.32 ± 0.30 | 0.0170 ± 0.0026 | 5.86 | 8.33 | 5.37 | 0.20 | 0 | 2.13 | 0.63 |
| 142 | 68 | Female | 3.42 ± 0.38 | 0.0150 ± 0.0017 | 4.27 | 4.51 | 2.57 | 0.05 | 0.04 | 1.60 | 0.25 |
| 143 | 69 | Male | 3.44 ± 0.16 | 0.0148 ± 0.0010 | 4.86 | 7.42 | 4.57 | 0.43 | 0.09 | 1.85 | 0.48 |
| 144 | 70 | Male | 2.99 ± 0.01 | 0.0117 ± 0.0004 | 5.18 | 6.20 | 3.81 | 0.26 | 0.01 | 1.68 | 0.44 |
| 145 | 71 | Male | 3.13 ± 0.20 | 0.0121 ± 0.0020 | 4.86 | 4.19 | 1.42 | 0.13 | 0.01 | 2.42 | 0.21 |
| 146 | 72 | Male | 3.00 ± 0.12 | 0.0109 ± 0.0009 | 4.41 | 4.76 | 3.09 | 0.11 | 0.04 | 1.14 | 0.37 |
| 147 | 73 | Male | 3.11 ± 0.30 | 0.0106 ± 0.0003 | 4.73 | 6.16 | 2.81 | 0.59 | 0.08 | 2.34 | 0.34 |
| 148 | 73 | Female | 2.91 ± 0.06 | 0.0122 ± 0.0001 | 5.16 | 6.00 | 3.85 | 0.17 | 0.05 | 1.44 | 0.49 |
| 149 | 74 | Male | 3.35 ± 0.05 | 0.0114 ± 0.0004 | 4.87 | 6.34 | 3.90 | 0.29 | 0.06 | 1.61 | 0.48 |
| 150 | 74 | Female | 2.92 ± 0.14 | 0.0123 ± 0.0006 | 3.72 | 4.77 | 3.10 | 0.06 | 0.03 | 1.17 | 0.41 |
| 151 | 75 | Male | 2.85 ± 0.12 | 0.0105 ± 0.0004 | 4.25 | 4.32 | 2.76 | 0.05 | 0.02 | 1.14 | 0.34 |
| 152 | 75 | Female | 3.43 ± 0.01 | 0.0122 ± 0.0011 | 4.72 | 6.97 | 3.78 | 0.23 | 0.03 | 2.14 | 0.78 |
| 153 | 76 | Male | 3.25 ± 0.08 | 0.0108 ± 0.0008 | 4.43 | 5.67 | 3.65 | 0.18 | 0.03 | 1.43 | 0.38 |
| 154 | 76 | Female | 3.08 ± 0.12 | 0.0111 ± 0.0005 | 4.33 | 5.72 | 3.42 | 0.16 | 0.04 | 1.83 | 0.27 |
| 155 | 77 | Male | 2.93 ± 0.10 | 0.0096 ± 0.0003 | 5.09 | 6.40 | 3.70 | 0.11 | 0.08 | 2.04 | 0.47 |
| 156 | 77 | Female | 2.78 ± 0.03 | 0.0125 ± 0.0013 | 4.73 | 5.53 | 3.59 | 0.32 | 0.03 | 1.17 | 0.43 |
| 157 | 78 | Male | 2.86 ± 0.06 | 0.0110 ± 0.0001 | 4.62 | 4.80 | 2.53 | 0.05 | 0.03 | 1.82 | 0.37 |
| 158 | 78 | Female | 2.74 ± 0.04 | 0.0111 ± 0.0007 | 3.70 | 3.94 | 2.04 | 0.02 | 0.01 | 1.57 | 0.31 |
| 159 | 79 | Male | 3.03 ± 0.01 | 0.0128 ± 0.0000 | 4.87 | 5.64 | 3.78 | 0.13 | 0.03 | 1.27 | 0.43 |
| 160 | 79 | Female | 3.00 ± 0.01 | 0.0131 ± 0.0011 | 4.25 | 6.17 | 3.81 | 0.13 | 0.08 | 1.74 | 0.42 |
| 161 | 80 | Male | 2.76 ± 0.00 | 0.0093 ± 0.0000 | 4.49 | 4.80 | 2.48 | 0.08 | 0.03 | 1.72 | 0.49 |
| 162 | 81 | Male | 3.36 ± 0.08 | 0.0094 ± 0.0005 | 4.70 | 9.30 | 5.25 | 0.38 | 0.07 | 3.04 | 0.55 |
| 163 | 81 | Female | 2.62 ± 0.08 | 0.0099 ± 0.0011 | 4.18 | 5.47 | 3.41 | 0.14 | 0.04 | 1.62 | 0.27 |
| 164 | 82 | Male | 3.20 ± 0.09 | 0.0092 ± 0.0012 | 4.17 | 7.12 | 4.06 | 0.07 | 0.04 | 2.45 | 0.50 |
| 165 | 83 | Male | 2.87 ± 0.04 | 0.0088 ± 0.0002 | 4.54 | 7.29 | 4.79 | 0.09 | 0.03 | 1.92 | 0.47 |
| 166 | 84 | Male | 2.78 ± 0.04 | 0.0104 ± 0.0018 | 3.94 | 6.57 | 3.57 | 0.24 | 0.06 | 2.14 | 0.56 |
| 167 | 85 | Female | 3.29 ± 0.04 | 0.0100 ± 0.0002 | 4.08 | 8.08 | 5.94 | 0.15 | 0.05 | 1.41 | 0.54 |
| 168 | 85 | Male | 3.20 ± 0.16 | 0.0094 ± 0.0003 | 5.12 | 7.64 | 4.06 | 0.2 | 0.02 | 2.82 | 0.54 |
| 169 | 86 | Male | 2.96 ± 0.00 | 0.0088 ± 0.0007 | 4.18 | 6.24 | 3.06 | 0.05 | 0.02 | 2.74 | 0.36 |
| 170 | 84 | Male | 2.65 ± 0.00 | 0.0106 ± 0.0002 | 3.96 | 5.11 | 2.99 | 0.04 | 0.02 | 1.53 | 0.54 |
| 171 | 88 | Male | 2.82 ± 0.07 | 0.0107 ± 0.0017 | 4.74 | 4.62 | 2.97 | 0.02 | 0.04 | 1.19 | 0.41 |
| 172 | 88 | Female | 3.18 ± 0.14 | 0.0103 ± 0.0005 | 3.36 | 8.21 | 4.51 | 0.24 | 0.11 | 2.65 | 0.70 |

**Supplemental Data Table S8.** The Spearman correlation of the contents of 5-mC and 5-hmC with respect to blood cell composition. RBC, red blood cells; WBC, white blood cells; NEUT, neutrophil granulocytes; EO, eosinophile granulocytes; BASO, basophile granulocytes; LYMPH, lymphocytes; MONO, monocytes.

|  | 5-mC | | 5-hmC | |
| --- | --- | --- | --- | --- |
|  | Spearman correlation coefficient | *p* value | Spearman correlation coefficient | *p* value |
| RBC | 0.029 | 0.706 | -0.031 | 0.687 |
| WBC | 0.257 | 0.0007 | 0.241 | 0.001 |
| NEUT | 0.167 | 0.029 | 0.223 | 0.003 |
| EO | 0.089 | 0.248 | -0.028 | 0.712 |
| BASO | 0.131 | 0.087 | -0.151 | 0.048 |
| LYMPH | 0.319 | 0.00002 | 0.233 | 0.002 |
| MONO | 0.058 | 0.449 | -0.084 | 0.271 |

**Supplemental Data Table S9.** The qualitative and quantitative ions for the detection of nucleosides.

| **Analytes** | **Charge state** | **Qualitative ions (*m/z*)** | **Quantitative ion (*m/z*)** |
| --- | --- | --- | --- |
| **A** | +1 | 268.103, 136.061 | 136.061 |
| **U** | +1 | 245.079, 113.036 | 113.036 |
| **C** | +1 | 244.092, 112.050 | 112.050 |
| **G** | +1 | 284.098, 152.065 | 152.065 |
| **dA** | +1 | 252.110, 136.061 | 136.061 |
| **T** | +1 | 243.098, 127.051 | 127.051 |
| **dC** | +1 | 228.098, 112.050 | 112.050 |
| **dG** | +1 | 268.105, 152.065 | 152.065 |
| **5-mdC** | +1 | 242.114, 126.068 | 126.068 |
| **5-hmdC** | +1 | 258.109, 142.062 | 142.062 |


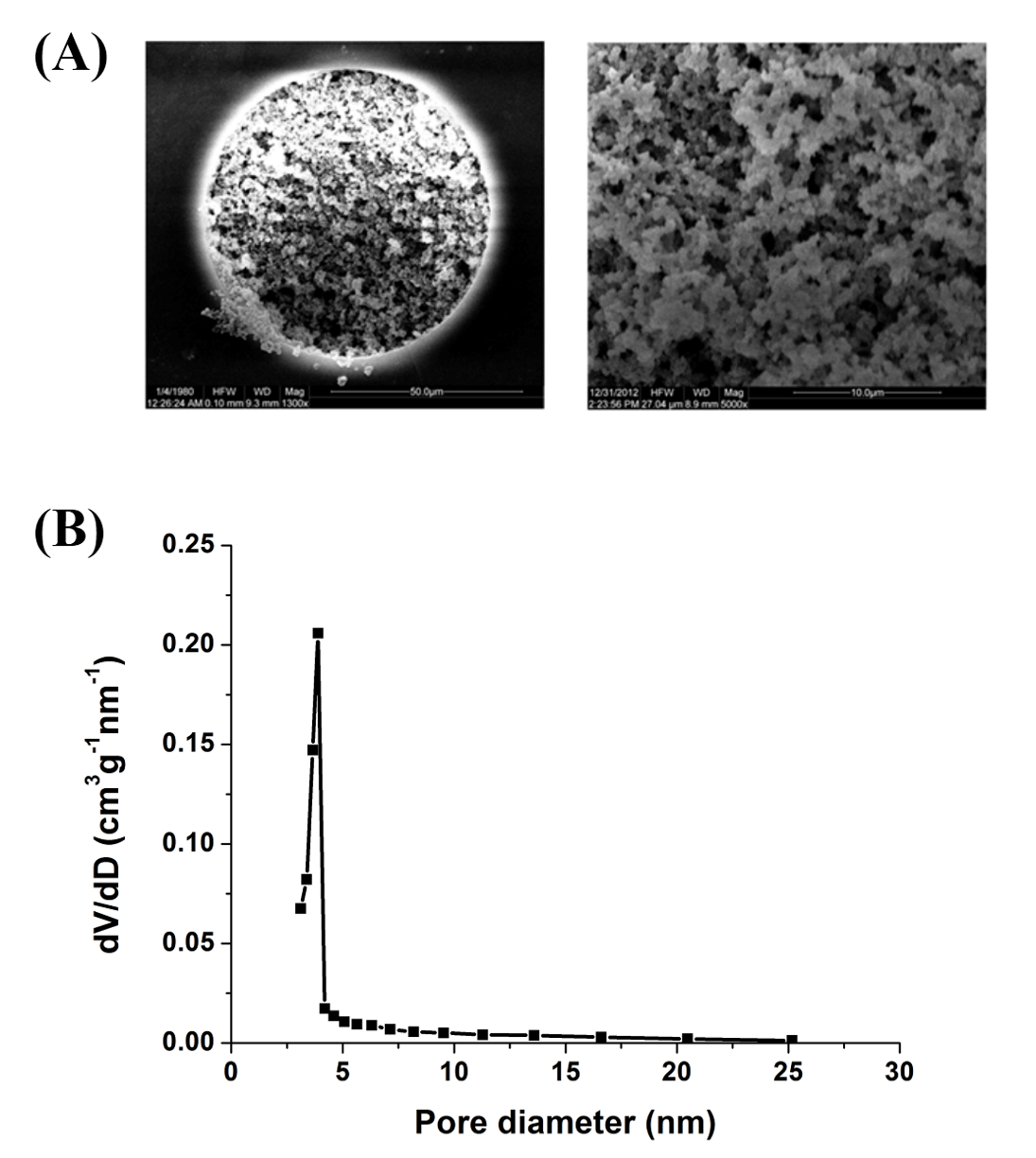


**Supplemental Data Figure S1.** Characterizations of hydrophilic organic-silica hybrid monolith. (A) Scanning electron microscope images of hydrophilic organic-silica hybrid monolith. Left, 1300 × wide-view; right, 5000 × close-up-view. (B) Mesoporous distribution of the hydrophilic organic-silica hybrid monolith.


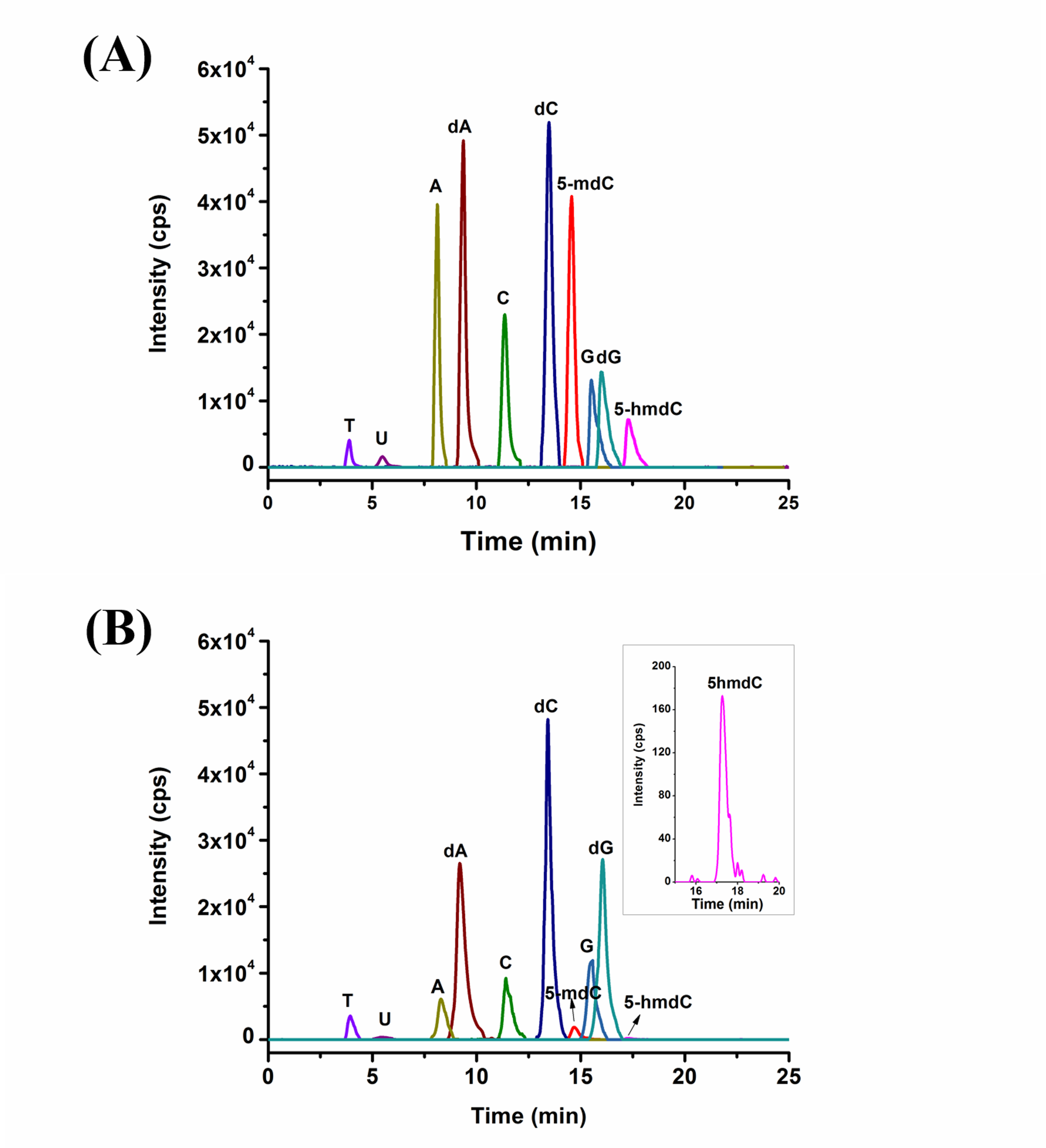


**Supplemental Data Figure S2.** Extracted-ion chromatograms of nucleosides by on-line trapping/*c*HILIC/ESI-MS analysis. (A) Nucleoside standards obtained under the optimized conditions. (B) Nucleosides from genomic DNA of blood (No. 147). Shown in the inset is the expanded chromatogram 5-hmdC.


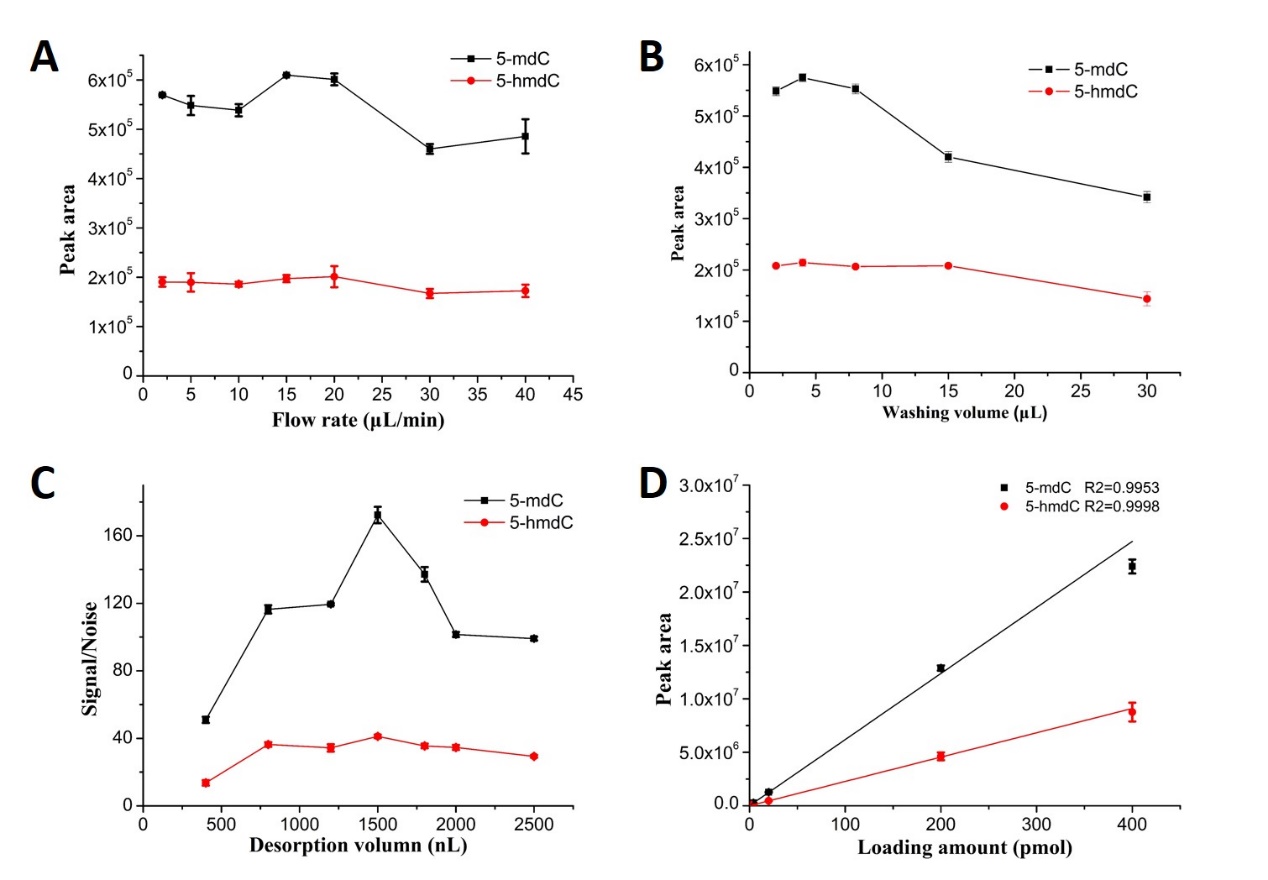


**Supplemental Data Figure S3.** Optimizations of the on-line trapping/*c*HILIC/ESI-MS conditions. The effects of (A) flow rate, (B) washing volume, (C) desorption volume and (D) loading amount on the analysis of 5-mdC and 5-hmdC by on-line trapping/*c*HILIC/ESI-MS method.


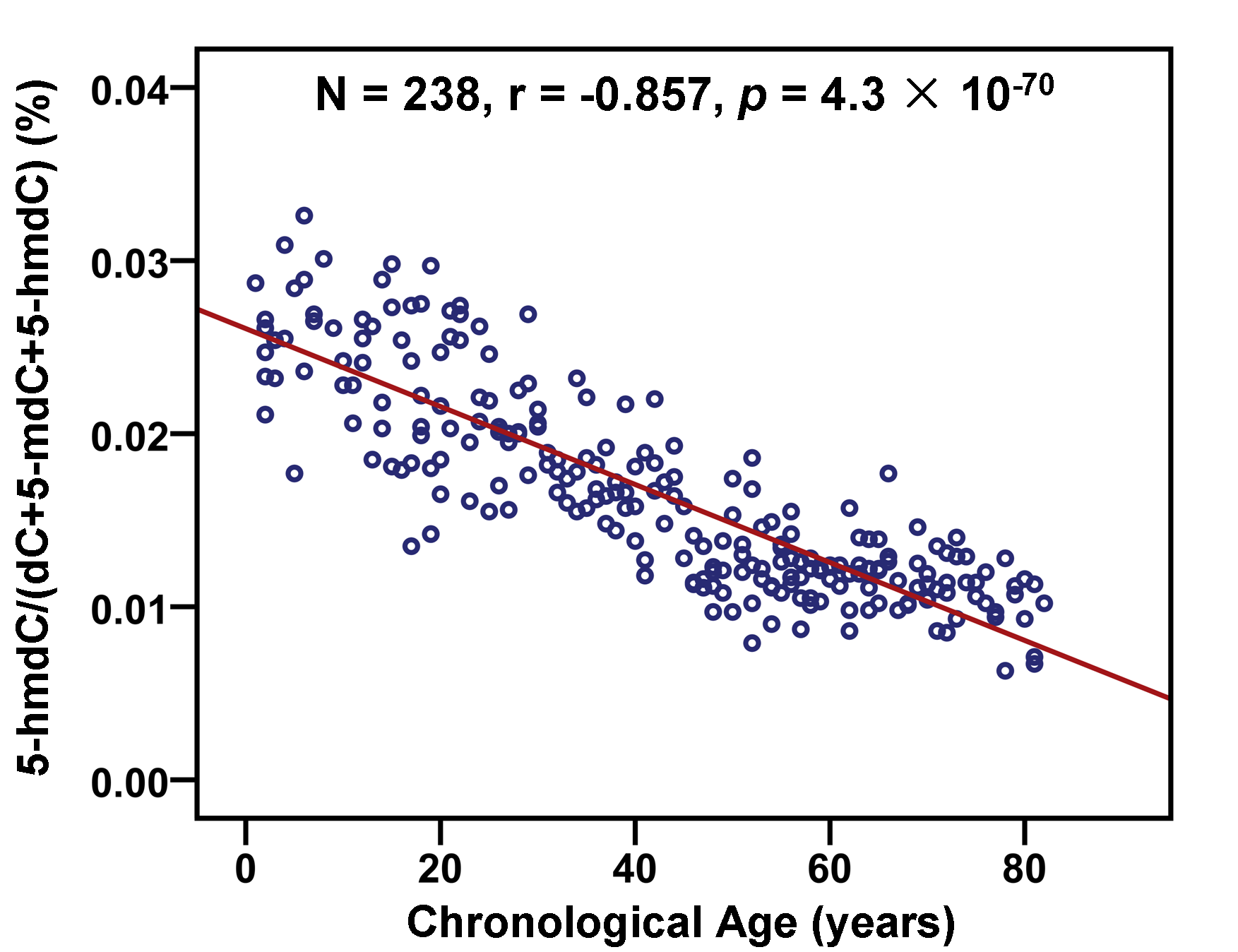


**Supplemental Data Figure S4.** The linear regression of 5-hmC content in genomic DNA of blood with age. The equation of the linear regression is “y = 0.02607 – 0.000225x”, where y represents 5-hmC content in genomic DNA and x represents chronological age.


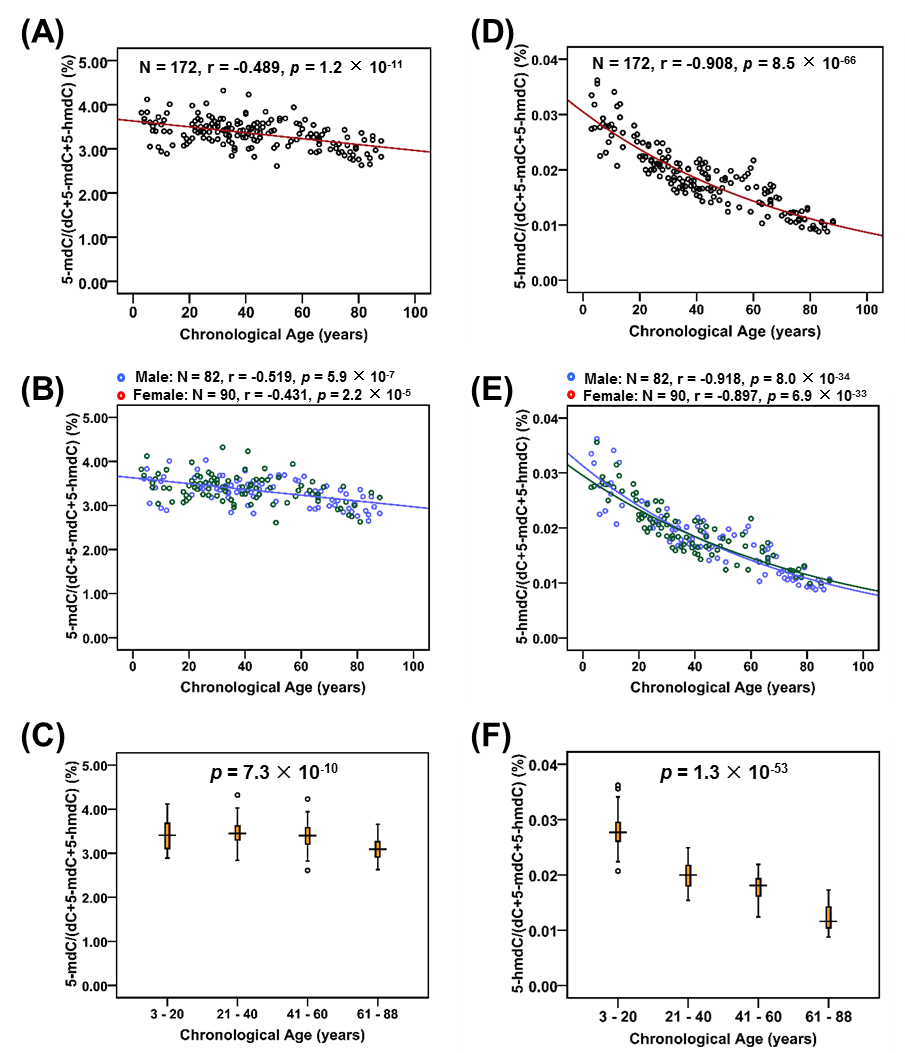


**Supplemental Data Figure S5.** Correlation analysis of DNA methylation and hydroxymethylation with age using blood samples from Henan province, China. A total of 172 samples including 72 males and 90 females aged from 3 to 88 years old were included in this study. (A) Exponential regression of 5-mC content in genomic DNA of blood with age. (B) Exponential regression of 5-mC content in genomic DNA of blood with age using different genders. (C) Comparison of 5-mC content at different age stages. (D) Exponential regression of 5-hmC content in genomic DNA of blood with age. The equation of the exponential regression is “y = 0.03047e^-0.01258x^”, where y represents 5-hmC content in genomic DNA and x represents chronological age. (B) Exponential regression of 5-hmC content in genomic DNA of blood with age using different genders. (C) Comparison of 5-hmC content at different age stages. Each point represents the 5-mC or 5-hmC content in one sample.


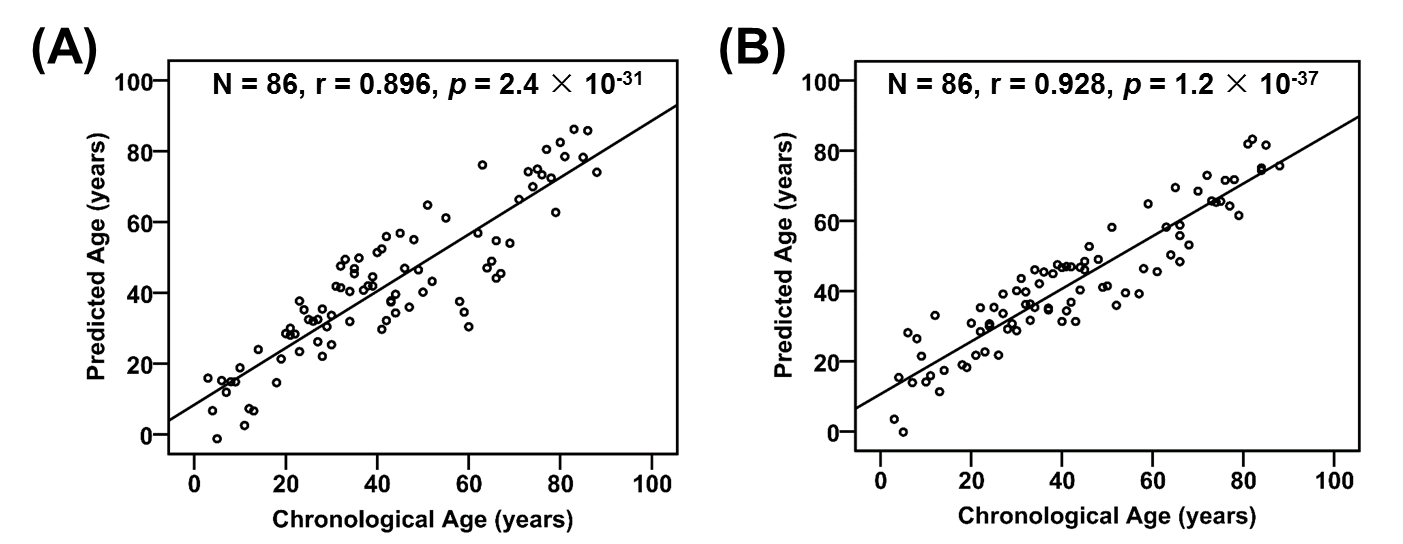


**Supplemental Data Figure S6.** Chronological age (x-axis) versus DNA hydroxymethylation age in the training group (A) and test group (B) using samples from Henan province, China.


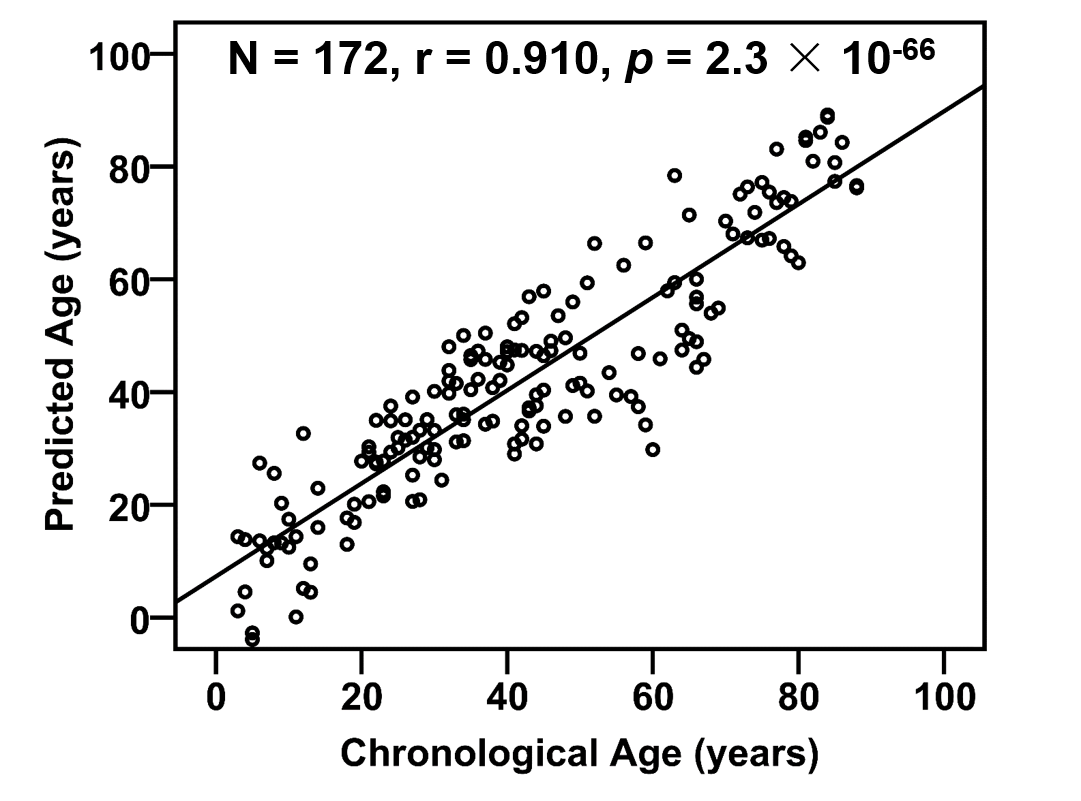


**Supplemental Data Figure S7.** Predicted age versus chronological age of all subjects using a leave-one-out model using samples from Henan province, China. A multivariate regression model was fit on all but one sample and its predicted age (y-axis) was related to the chronological age of the left out sample (x-axis).


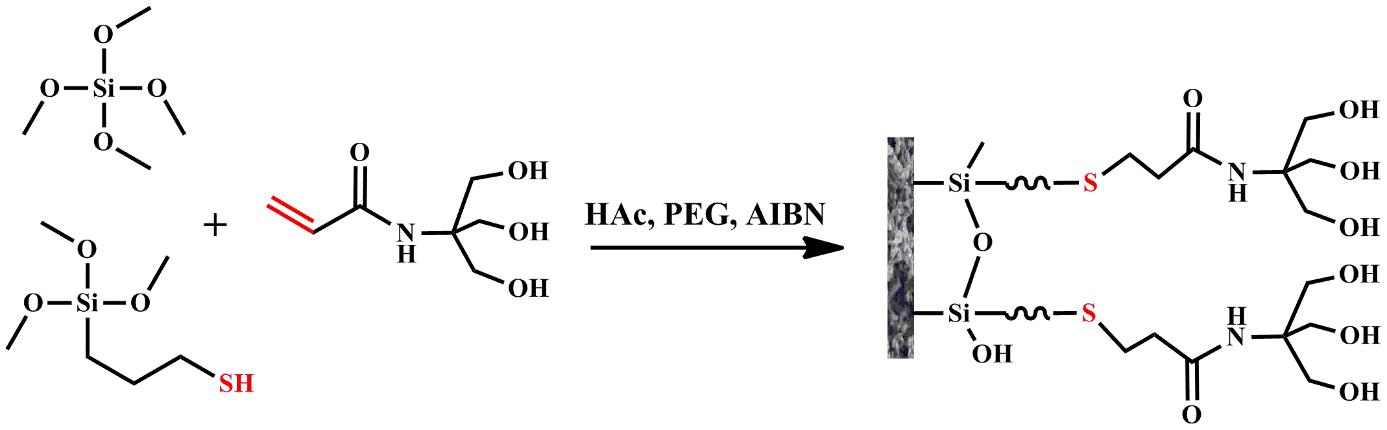


**Supplemental Data Figure S8.** Schematic procedure for the preparation of hydrophilic organic-silica hybrid monolith by sol-gel method combined with ‘‘thiol-ene’’ click reaction in “one-pot”.
